# Thiourea Derivative of 2-[(1*R*)-1-Aminoethyl]phenol: A Flexible Pocket-like Chiral Solvating Agent (CSA) for the Enantiodifferentiation of Amino Acid Derivatives by NMR Spectroscopy

**DOI:** 10.1021/acs.joc.0c00027

**Published:** 2020-03-19

**Authors:** Alessandra Recchimurzo, Cosimo Micheletti, Gloria Uccello-Barretta, Federica Balzano

**Affiliations:** Department of Chemistry and Industrial Chemistry, University of Pisa, via Moruzzi 13, 56124 Pisa, Italy

## Abstract

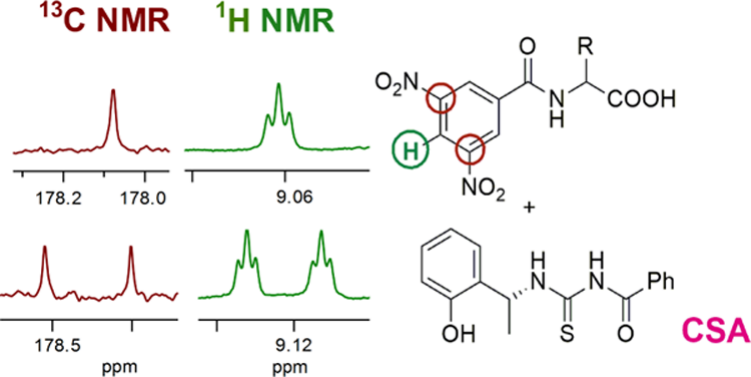

Thiourea derivatives of 2-[(1*R*)-1-aminoethyl]phenol, (1*S*,2*R*)-1-amino-2,3-dihydro-1*H*-inden-2-ol, (1*R*,2*R*)-(1*S*,2*R*)-1-amino-2,3-dihydro-1*H*-inden-2-ol, and (*R*)-1-phenylethanamine have been compared as chiral solvating agents (CSAs) for the enantiodiscrimination of derivatized amino acids using nuclear magnetic resonance (NMR) spectroscopy. Thiourea derivative, prepared by reacting 2-[(1*R*)-1-aminoethyl]phenol with benzoyl isothiocyanate, constitutes an effective CSA for the enantiodiscrimination of *N-*3,5-dinitrobenzoyl (DNB) derivatives of amino acids with free or derivatized carboxyl functions. A base additive 1,4-diazabicyclo[2.2.2]octane(DABCO)/*N*,*N*-dimethylpyridin-4-amine (DMAP)/NBu_4_OH) is required both to solubilize amino acid derivatives with free carboxyl groups in CDCl_3_ and to mediate their interaction with the chiral auxiliary to attain efficient differentiation of the NMR signals of enantiomeric substrates. For ternary systems CSA/substrate/DABCO, the chiral discrimination mechanism has been ascertained through the NMR determination of complexation stoichiometry, association constants, and stereochemical features of the diastereomeric solvates.

## Introduction

In the continuous search for new efficient and direct methods of chiral analysis, NMR spectroscopy offers several opportunities based on the use of chiral auxiliaries able to transfer enantiomers in a diastereomeric environment, thus generating differentiation of their observable NMR parameters. Some chiral auxiliaries, which are named chiral derivatizing agents (CDAs),^1−3^ are employed for the chemical derivatization of the two enantiomers, via the formation of covalent linkages. Chiral solvating agents (CSAs, diamagnetic)^3−5^ and chiral lanthanide shift reagents (CLSRs, paramagnetic)^3,6^ are, instead, simply mixed with the enantiomeric substrates and, based on noncovalent intermolecular interactions in solution, diastereomeric solvates or complexes are formed directly in the NMR tube. The use of diamagnetic CSAs has emerged as particularly convenient from the practical point of view: 1 or 2 equiv of the suitable CSA is added directly to the NMR tube and corresponding signals of the two enantiomers can be easily differentiated and identified in the NMR spectrum without significant line-broadening effects, as in the case of paramagnetic CLSRs. The prominent role of CSAs is clearly witnessed by the flourishing literature dedicated to this class of chiral auxiliaries for NMR spectroscopy, spanning from the milestone Pirkle’s alcohol first proposed in 1977^7^ to highly preorganized complex structures.^3−5^ In particular, thiourea^8^ and bisthiourea^9−13^ CSAs have been proposed for the NMR analyses of chiral anionic substrates, such as α-hydroxy and α-aminocarboxylates. Preformed carboxylates can be analyzed as in the case of tetrabutylammonium salts,^8,12,13^ or the use of strong bases such as 1,4-diazabicyclo[2.2.2]octane (DABCO) or *N*,*N*-dimethylpyridin-4-amine (DMAP)^9−11^ has been suggested to mediate the interaction between the carboxylic acid and the CSAs.

**Figure 1:**
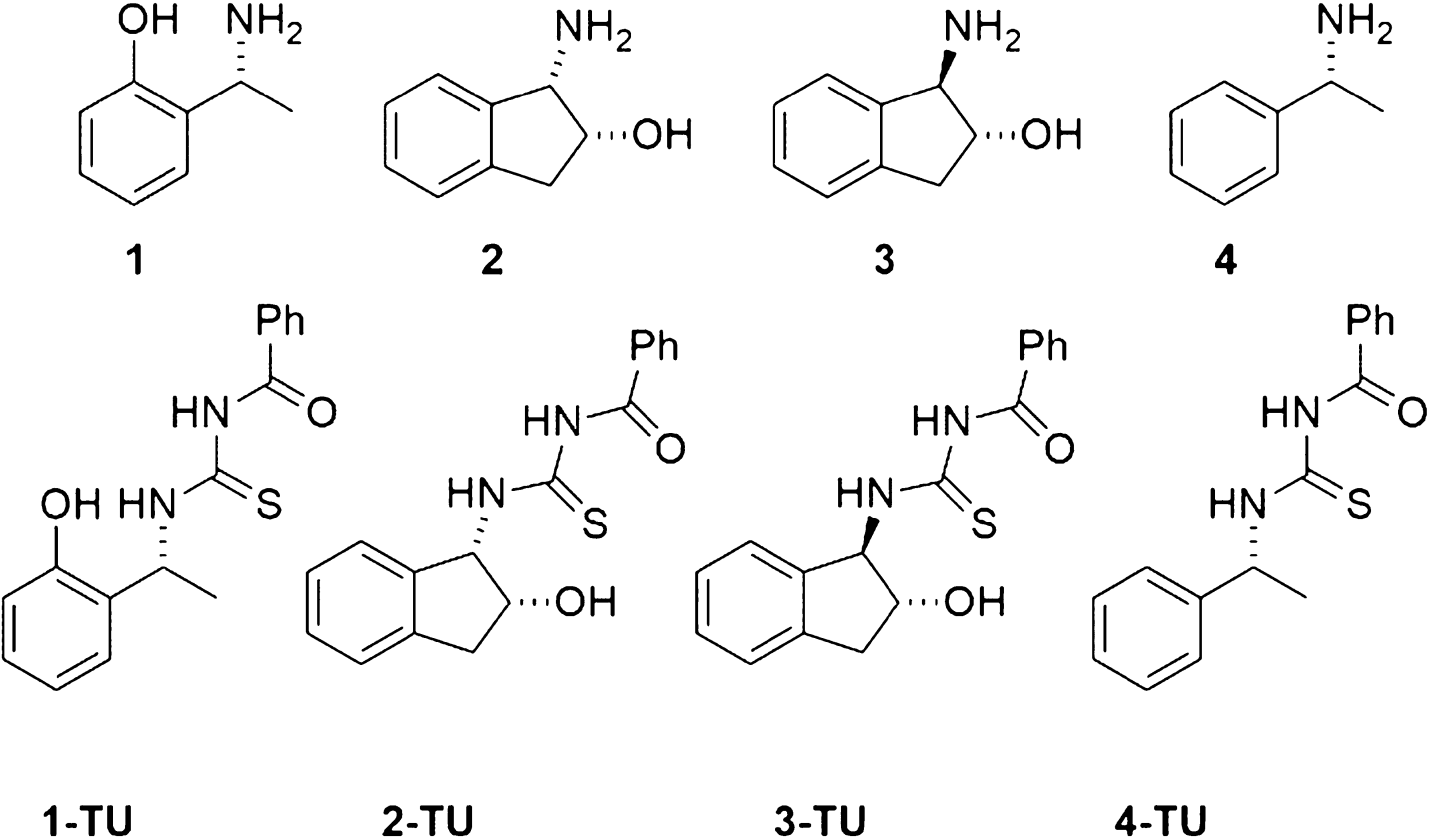
CSA structures.

In consideration of their potentialities as efficient and versatile CSAs for NMR spectroscopy, we focused on thiourea derivatives **1-TU**, **2-TU**, **3-TU**, and **4-TU** of commercially available amino alcohols **1**–**3** and amine **4** (Figure 1), the amino groups of which were selectively and quantitatively derivatized by the reaction with benzoyl isothiocyanate. Compound **1** is endowed with an acidic phenolic hydroxyl, which was not present in **4**. Compounds **2** and **3** have rigid structures with cis and trans amino and hydroxy groups, respectively.

**Figure 2:**
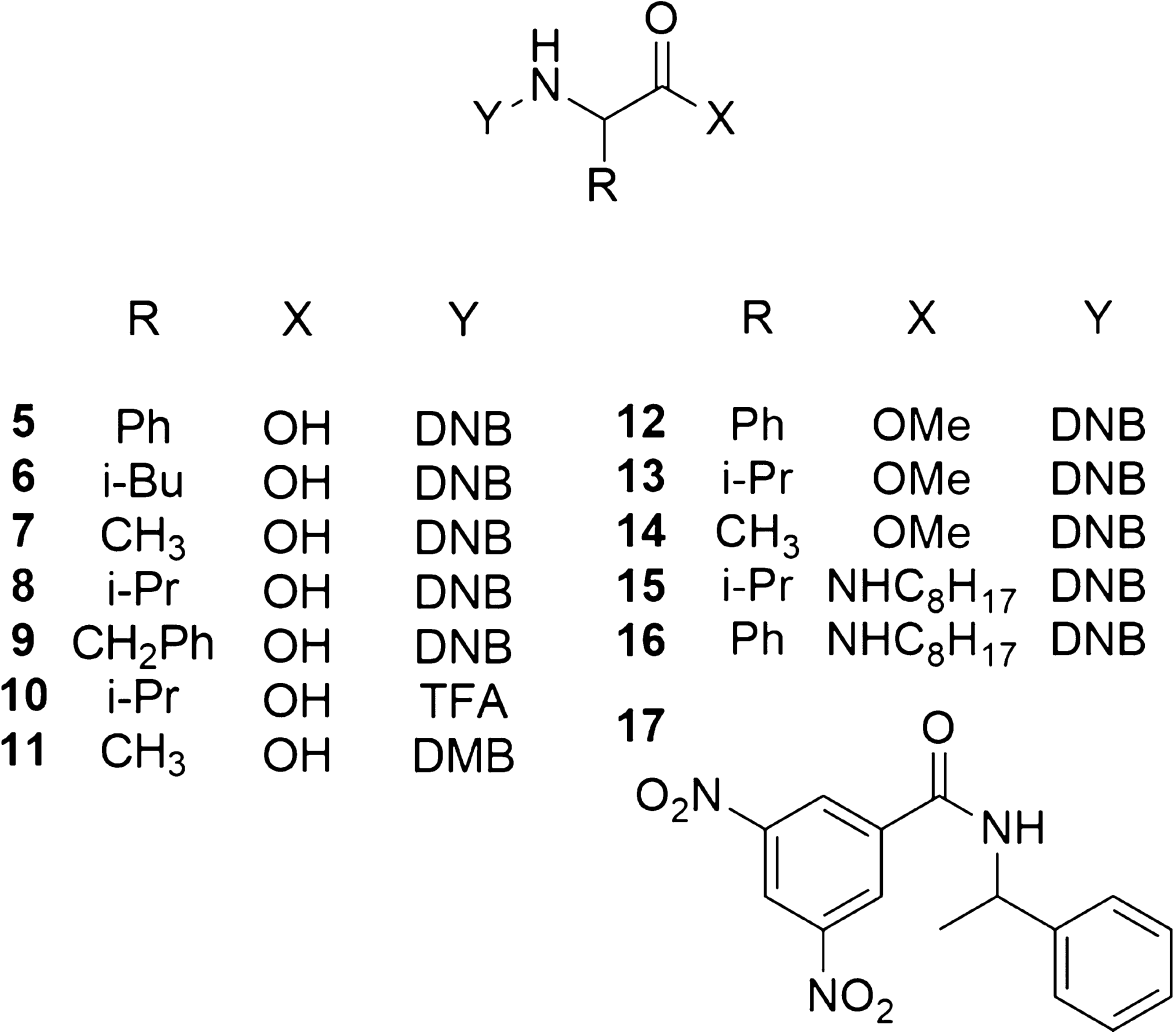
Chemical structures of derivatives **5**–**17** (DNB = 3,5-dinitrobenzoyl, TFA = trifluoroacetyl, DMB = 3,5-dimethoxybenzoyl).

The efficiency of CSAs has been compared in multinuclear NMR enantiodiscrimination experiments of several kinds of amino acid derivatives (Figure 2), including N-derivatives of α-amino acids with free carboxyl groups, and π-acceptor (**5**–**9**) or π-donor (**11**) aromatic moieties. Compound **10** is endowed with a fluorinated probe for enantiodifferentiation by ^19^F NMR. Derivatization as in the case of compounds **12**–**16** allowed to evaluate the contribution of the carboxyl group to enantiodifferentiation. A base (DABCO, DMAP, NBu_4_OH) is required to solubilize the amino acid derivatives **5**–**11** in CDCl_3_.

With the aim of gaining insight into the origin of enantiodiscrimination processes, NMR investigations have been carried out on the stereochemical and thermodynamic features of the diastereomeric solvates formed by a selected CSA and the enantiomeric substrates.

## Results and Discussion

Chiral auxiliaries **1-TU**, **2-TU**, **3-TU**, and **4-TU** were obtained in a quantitative yield by reacting commercially available amino alcohols **1**–**3** and amine **4** (Figure 1) with benzoyl isothiocyanate. CSAs were characterized by the use of two-dimensional (2D) NMR techniques (see the Experimental Section).

### ^1^H NMR Enantiodiscrimination

Enantiodiscrimination efficiencies both of thiourea derivatives and their amino alcohol precursors were evaluated in the NMR spectra by measuring the nonequivalence (ΔΔδ = |Δδ_R_ – Δδ_S_|, where Δδ_R_ = δ_mixture_^R^ – δ_free_ and Δδ_S_ = δ_mixture_^S^ – δ_free_), i.e., the magnitude of the splitting of corresponding resonances of the enantiomeric substrate in its mixture containing CSAs. Underivatized amino acids were not considered because of their low solubility in CDCl_3_ or DMSO-*d*_6_, also in the presence of the base. Their derivatives **5**–**16** were analyzed, among which compounds **5**–**11** were solubilized in CDCl_3_ by adding one equivalent of the base (DABCO/DMAP/NBu_4_OH). Compounds **12**–**17** are completely soluble in CDCl_3_ and employed without a base.

Amino alcohols **1**–**3** showed very poor solubility in CDCl_3_ and enantiodiscrimination experiments could be carried out only in DMSO-*d*_6_ or mixtures CDCl_3_/DMSO-*d*_6_ containing the minimum amount of DMSO-*d*_6_ needed to solubilize the CSA. In such conditions, however, any doublings of NMR signals of amino acid derivatives were not detected. Amine **4** is soluble in CDCl_3_, but slow-exchange processes between its protonated and unprotonated forms were detected in the NMR spectra in the presence of amino acids with free carboxyl groups, pure or premixed with DABCO.

**Figure 3:**
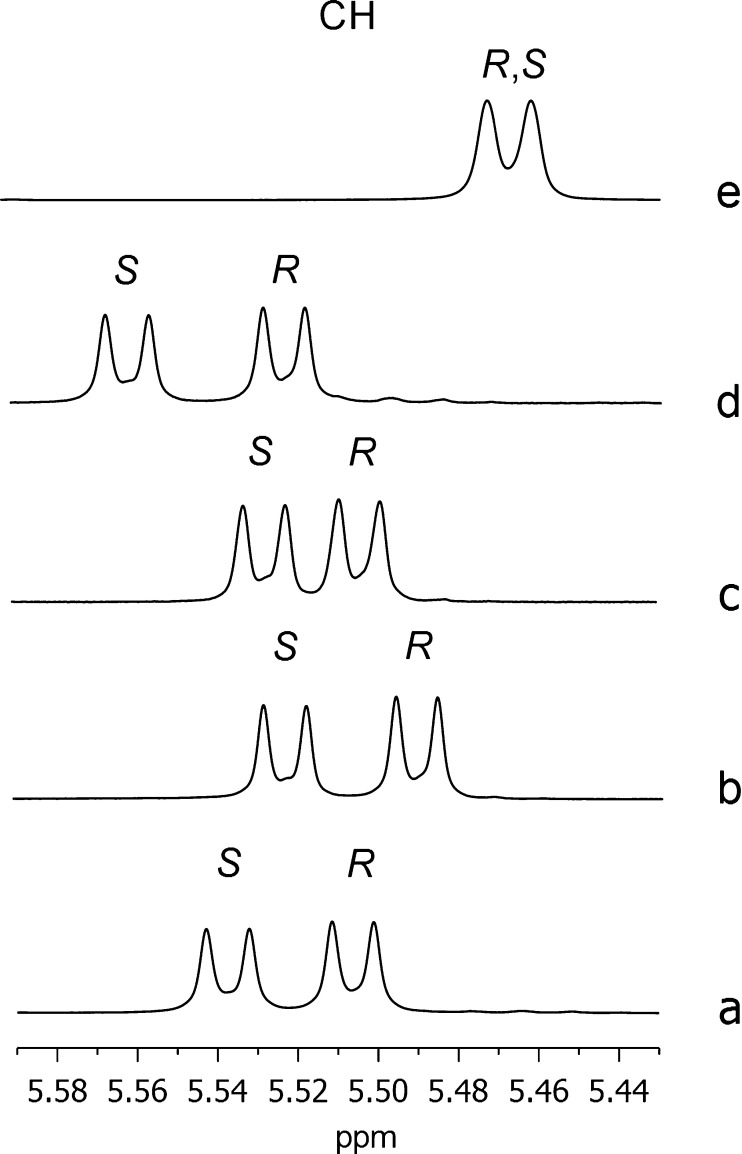
^1^H NMR (600 MHz, CDCl_3_, 25 °C) spectral regions corresponding to the aliphatic methine protons of (*R*,*S*)-**5** in **1-TU**/DABCO/**5** (1:1:1) mixtures: [**5**] = 60 mM (a), 30 mM (b), and 15 mM (c), in the **1-TU**/DABCO/**5** (2:1:1) mixture ([**5**] = 15 mM) (d), and in the DABCO/**5** (1:1) mixture ([**5**] = 30 mM) (e).

Thiourea derivatives **1-TU**, **2-TU**, **3-TU**, and **4-TU** are all soluble in CDCl_3_. Adding 1 equiv of **1-TU** to the equimolar mixture **5**/DABCO in CDCl_3_ produced the same amount of enantiomer differentiation as in the case of the addition of 1 equiv of DABCO to the equimolar mixture **1-TU**/**5**. At 60 mM, very high nonequivalences of 0.147 and 0.090 ppm were measured for the ortho and para protons of the 3,5-dinitrobenzoyl moiety, respectively (Table 1). Lower, but still relevant, doublings of 0.019 and 0.031 ppm were measured at the NH and CH protons, respectively (Table 1). Even higher nonequivalences were measured in the mixture containing 60 mM **1-TU** and 30 mM **5**/DABCO (Table 1). On changing both the CSA concentration and the CSA to substrate molar ratio, appreciable differentiation of enantiotopic nuclei was obtained up to 5 mM equimolar amounts of CSA and **5**/DABCO (Figure 3 and Table 1).

**Table 1:** ^1^H NMR (600 MHz, CDCl_3_, 25 °C) Nonequivalences (ppm) for **5** in the Presence of 1 equiv of DABCO in **5**/DABCO/**1-TU** Mixtures

[**5**]	60 mM	30 mM		15 mM				5 mM			
**5**/**1-TU**	1:1	1:1	1:2	1:1	1:2	1:3	1:4	1:1	1:2	1:3	1:4
*o*-DNB	0.147	0.129	0.203	0.094	0.156	0.193	0.224	0.027	0.044	0.077	0.097
*p*-DNB	0.090	0.079	0.124	0.058	0.096	0.112	0.135	0.016	0.029	0.048	0.061
NH	0.019	0.010	0.032	0.024	0.045	0.057	0.066	0.014	0.037	0.052	0.066
CH	0.031	0.034	0.049	0.024	0.039	0.047	0.053		0.005	0.010	0.012

Different base additives were probed in the mixture **5**/**1-TU**, among which DMAP was comparable to DABCO for the ortho and para protons of the 3,5-dinitrobenzoyl moiety; lower nonequivalences were detected for the methine proton and a better result for the NH group (Table 2). The effect of **1-TU** on the tetrabutylammonium salt of **5** was quite similar to those of **5**/DABCO and **5**/DMAP mixtures, to indicate a scarce dependence on the nature of the base additive. However, on considering the spectral features of the three bases, DABCO was selected since its 12 isochronous protons originated a unique resonance centered at 2.78 ppm.

**Table 2:** Effect of 1 equiv of Base on ^1^H NMR (600 MHz, CDCl_3_, 25 °C) Nonequivalences (ppm) for **5** (30 mM) in the Presence of 1 equiv of **1-TU**

	base		
proton	DABCO	DMAP	NBu_4_^+^
*o*-DNB	0.129	0.140	0.088
*p*-DNB	0.079	0.081	0.052
NH	0.010	0.040	0.028
CH	0.034	0.009	0.022

To ascertain the role of the base additive in the enantiodiscrimination processes, beyond its solubilizing efficacy, the equimolar mixture **1-TU**/**5** (without base additive) was analyzed in CDCl_3_ containing the minimum amount of DMSO-*d*_6_ (6% v/v) needed for the solubilization of the substrate: no significant doublings of **5** resonances were detected. By contrast, the addition of DABCO in the analogous solvent mixture of **1-TU**/**5** caused doublings, which were significant, even though lower than those in sole CDCl_3_ (Supporting Information, Figure S1). Therefore, the base additive plays a fundamental role in the enantiodiscrimination processes.

In consideration of the above-mentioned results, the experimental conditions of 30 mM CSA and 15 mM equimolar substrate/DABCO mixture were selected for the enantiodiscrimination experiments of substrates **5**–**11** (Table 3), where nonequivalences even higher than those obtained at the 60 mM equimolar conditions were measured (Table 1); therefore, lower amounts of CSA and substrate are required. Among **5**–**11**, the magnitude of enantiomers differentiation in the NMR spectra was quite similar for the 3,5-dinitrobenzoyl derivatives of phenylglycine **5**, leucine **6**, and alanine **7**, whereas lower values were measured in the cases of valine **8** and phenylalanine **9**.

**Table 3:** ^1^H NMR (600 MHz, CDCl_3_, 25 °C) Nonequivalences (ppm) of **5**–**16** (15 mM) in the Presence of **1-TU** (30 mM) and DABCO (15 mM) for **5**–**11**

substrate	*o-*DNB	*p*-DNB	NH	CH
**5**	0.156	0.096	0.045	0039
**6**	0.132	0.082	0.025	0.004
**7**	0.134	0.095	0.072	0.037
**8**	0.084	0.055	0.005	0.008
**9**	0.034	0.016	nd^a^	
**10**			nd^a^	0.006
**11**	0.005^b^	0.003^c^		
**12**	0.079	0.050	0.087	nd^a^
**13**	0.029	0.023	0.038	0.014
**14**	0.050	0.038	nd^a^	
**15**	0.069	0.035	0.011	0.011
**16**	0.138	0.078	0.033	

a nd = not determined.

b *o*-DMB.

c *p-*DMB.

The importance of the 3,5-dinitrobenzoyl moiety of the amino acid derivatives was demonstrated by comparison with compounds **10** and **11**, respectively, containing trifluoroacetyl and 3,5-dimethoxybenzoyl as derivatizing groups, for which no significant doublings of proton (or fluorine in the case of **10**) resonances were observed (Table 3).

Carboxyl group derivatization in the form of methyl esters (**12**–**14**) allowed to improve solubility in CDCl_3_ and DABCO was not required; however, under the same experimental conditions, nonequivalences were nearly half of the analogous derivatives with underivatized carboxyl groups (Table 3).

The presence of an additional NH group, as for derivatives **15** and **16** of valine and phenylglycine, led to obtaining nonequivalences analogous to those measured for the corresponding derivatives (**8** and **5**) with free carboxyl groups (Table 3).

The presence of the carboxyl function, derivatized or underivatized, is essential since, in the case of the 3,5-dinitrobenzoyl derivative of 1-phenylethanamine, **17** (Figure 2), no differentiation was produced by **1-TU**.

Therefore, it seems that derivatizing amino acids only at their amino groups by introducing a 3,5-dinitrobenzoyl moiety (**5**–**9**) represents the easiest and most effective way for the optimization of enantiomers differentiation by **1-TU**, provided that a base additive is employed.

Selected substrates (**5**, **6**, **12**, and **16**) were mixed with equimolar amounts (30 mM) of **2-TU**, **3-TU**, and of the thiourea derivative of 1-phenylethanamine, **4-TU**, having the same main skeleton of **1-TU**, but devoid of the phenolic hydroxyl. In all of the cases, enantiodifferentiation was less efficient than that in the presence of **1-TU** (Supporting Information, Table S1). As an example, **2-TU** caused very poor differentiation of the ortho protons of the 3,5-dinitrobenzoyl moiety and of the methine proton at the chiral center of **5**: 0.004 and 0.006 ppm, respectively, to be compared with 0.129 and 0.034 ppm measured in the presence of **1-TU**. An enantiodifferentiation of 0.028 ppm was observed for the para proton of the same moiety, almost a third of that measured in the presence of **1-TU**. The corresponding trans derivative, namely **3-TU**, did not produce any signal splitting of **5**, **6**, **12**, and **16**, even though the NH proton of **5** underwent a relevant line broadening and a low-frequency complexation shift (Δδ = −0.087 ppm, greater than those measured for the same proton in the presence of **2-TU** and **1-TU**, Supporting Information Table S2), which both suggest the binding ability of the chiral auxiliary. In the mixture **4-TU**/**5**/DABCO, protons of the 3,5-dinitrobenzoyl group were not differentiated at all and very low doublings of 0.005 ppm were obtained for the NH and CH protons. **4-TU** showed lower enantiodiscrimination efficiency even toward the leucine derivative **6**. For substrates **12** and **16** with derivatized carboxyl functions, nonequivalences between 0.001 and 0.004 ppm were measured, i.e., remarkably lower in comparison with the values obtained in the presence of **1-TU** (0.011–0.127 ppm) in the same experimental conditions (Supporting Information, Table S1). Therefore, the role of the phenolic hydroxy group is clearly demonstrated.

### ^13^C{^1^H} NMR Enantiodiscrimination

Among the NMR active nuclei, ^1^H is traditionally privileged in the detection of chiral discrimination phenomena by NMR: its high sensitivity arising both from the very high natural abundance and the high gyromagnetic ratio, as well as quite low recovery times of magnetization, allow to obtain quantitative results in reduced experimental times. As a counterpart of the above-mentioned advantages, ^1^H–^1^H scalar couplings and couplings with other nuclei with high natural abundance may produce complex multiplets, thus reducing the accuracy of the quantitative determinations. For this reason, several kinds of NMR experimental tools have been developed to suppress ^1^H–^1^H homonuclear couplings in spectra where the resonances of the two enantiomers are singlets, as in the case of “pure shift” experiments,^14,15^ which have become increasingly popular in chiral discrimination investigations. The observation of other high-sensitivity heteronuclei, such as ^19^F or ^31^P, has been frequently described.^16^ Detection of ^13^C nuclei, which are present in the molecular skeleton of all of the organic molecules, would be very attractive, but their low sensitivity has so far hampered this possibility. However, recent improvements in high-field NMR instruments have made accessible the observation of ^13^C nuclei at moderate concentrations and with reasonable experimental times.^4,16−20^ The advantages of observing ^13^C nuclei are that quaternary carbons, in addition to protonated ones, can be detected and carbon resonances of proton-decoupled spectra have very narrow line widths, which enable to perform accurate quantification of the two enantiomers even for small nonequivalences. One of the major concerns, which are raised about the observation of quaternary carbons, deals with the fact that they produce signals with reduced intensity in comparison with protonated carbons. However, in enantiodiscrimination experiments, we compare the integrated area of corresponding signals of the two enantiomers and diamagnetic CSAs usually affect line widths of the two enantiomers to the same extent.

**Figure 4:**
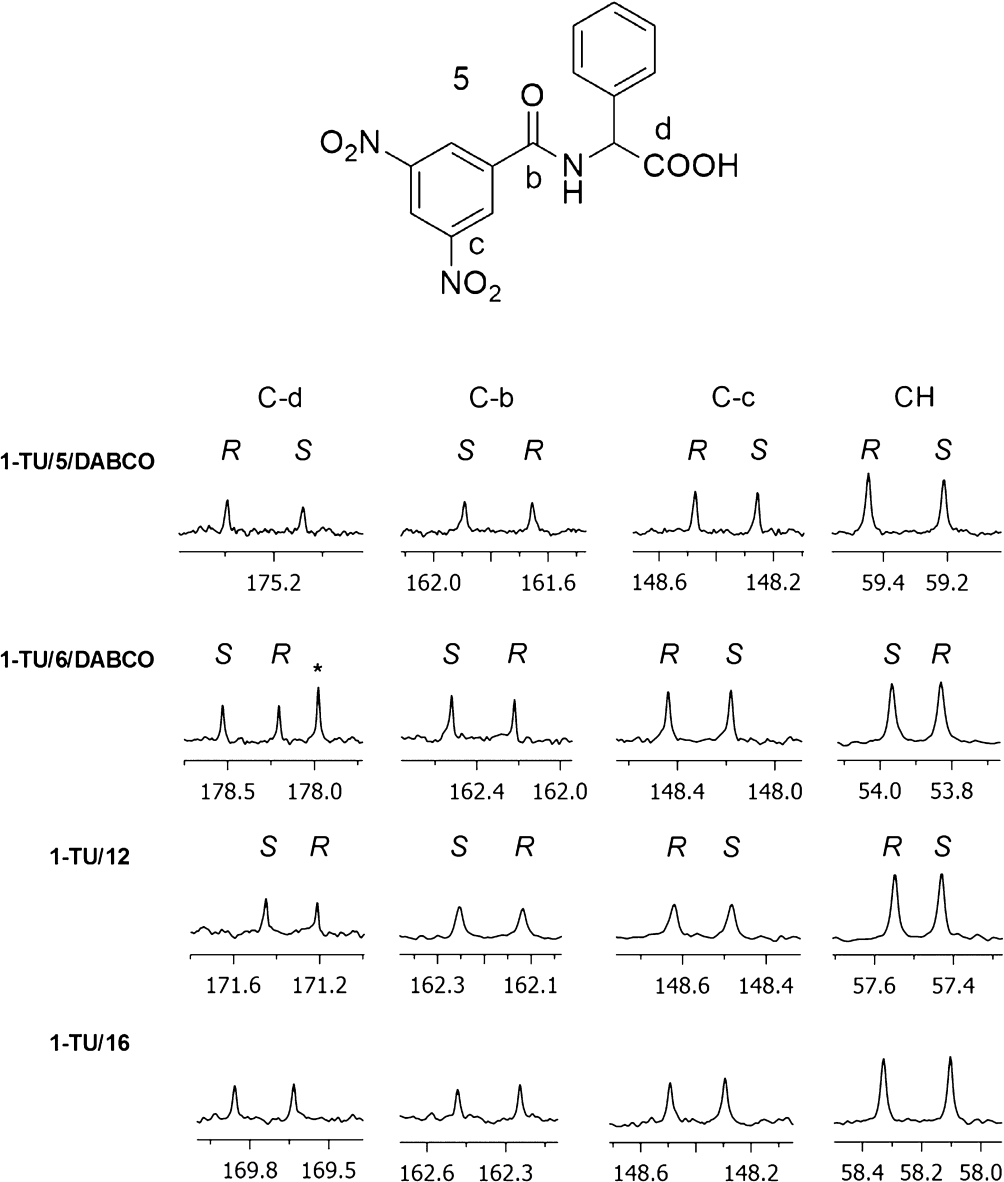
^13^C{^1^H} NMR (150 MHz, CDCl_3_, 25 °C) spectral regions corresponding to carboxy (C-d) and amide (C-b) carbonyl carbons, C-NO_2_ (C-c), and aliphatic methine (CH) of **5**, **6**, **12**, and **16** (30 mM) in the presence of 1 equiv of **1-TU** and 1 equiv of DABCO for **5** and **6**. *Resonance of CSA.

Therefore, we carried out ^13^C{^1^H} NMR enantiodiscrimination experiments of selected substrates (**5**, **6**, **12**, and **16**) (Figure 4 and Supporting Information Table S3).

In the equimolar mixture **1-TU**/**5**/DABCO (30 mM), very high differentiations of 0.313 and 0.241 ppm were obtained for the quaternary carbonyl carbons of carboxy (C-d) and amide (C-b) functions, respectively (Figure 4 and Supporting Information Table S3). Differentiation of quaternary carbons directly bound to the nitro groups (C-c) was similarly high and equal to 0.219 ppm. Among protonated carbons, the methine carbon at the chiral center of **5** was differentiated by 0.236 ppm (Figure 4 and Supporting Information Table S3). As shown in Table S3 (Supporting Information) and represented in Figure 4, correspondingly high nonequivalences were measured for the analogous derivative of leucine **6** with the free carboxyl function, and for the two derivatives **12** and **16**.

### Interaction Mechanism 1-TU/(*S*)-5 and 1-TU/(*R*)-**5**

**Figure 5:**
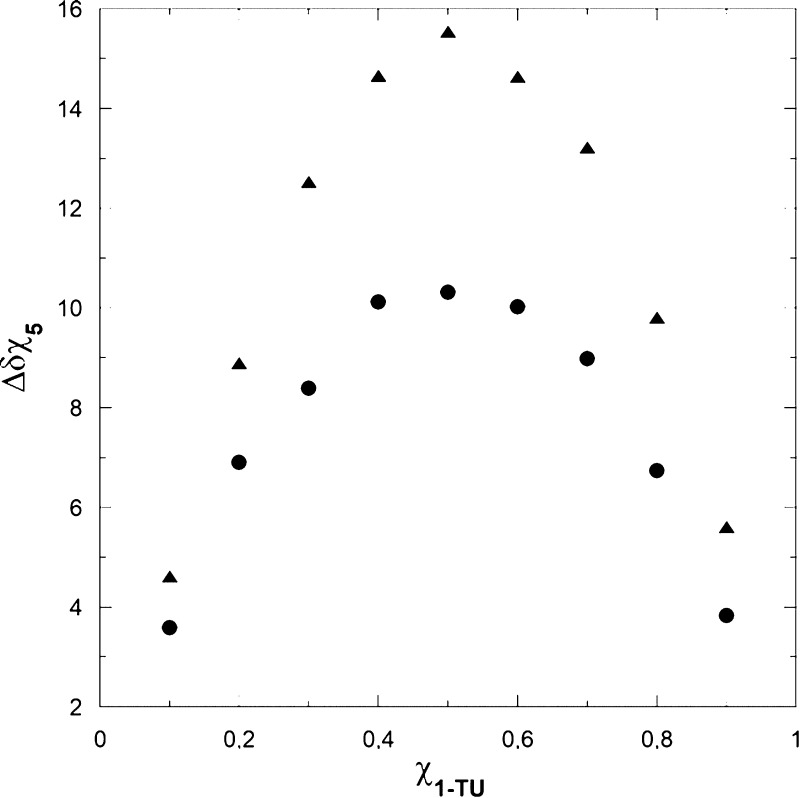
Stoichiometry determination based on ortho (▲) and para (●) protons of the DNB group for the (*S*)-**5**/**1-TU**/DABCO complex.

The complexation stoichiometries of the diastereomeric complexes **1-TU**/(*S*)-**5** and **1-TU**/(*R*)-**5** were defined using Job’s method.^21,22^ The chemical shifts of selected protons were measured in CDCl_3_ solutions of CSA/**5** mixtures at variable molar ratios and a constant total concentration (10 mM). By graphing the normalized complexation shifts of one component as a function of the molar fraction of the other one, bell curves were obtained with a well-defined maximum at 0.5 molar fraction corresponding to a 1-to-1 complexation stoichiometry (Figure 5).

The nonlinear fitting of dilution data^23^ (40/0.5 mM, Figure S2 in the Supporting Information) based on eq 1 gave the association constants (*K*) of the two diastereomeric complexes **1-TU**/(*S*)-**5** and **1-TU**/(*R*)-**5** (Table 4).
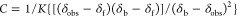
1where *C* is the molar concentration of CSA or substrate, δ_f_ and δ_b_ are the chemical shifts of the free and bound species, respectively, and δ_obs_ is the chemical shift measured for the selected proton in the equimolar mixture CSA/**5**.

**Table 4:** Association Constants (*K*, M^–1^) Obtained Using the Dilution Method for (*S*)-**5**/DABCO/**1-TU** and (*R*)-**5**/DABCO/**1-TU**

*K* (M^–1^)	
(*S*)-**5**/DABCO/**1-TU**	(*R*)-**5**/DABCO/**1-TU**
65.8 ± 5	30.2 ± 0.6

To ascertain the nature of the interactions that contribute to the stabilization of the two diastereomeric complexes, their stereochemistry was investigated by means of one-dimensional (1D) and 2D rotating-frame Overhauser (ROE) measurements, starting from the analysis of the conformation of pure **1-TU**.

As shown in Figure S3a (Supporting Information), proton NH(3) did not give ROE at the frequency of NH(2) and the magnitudes of inter-ROEs H_4_–H_5_ and H_4_–NH(3) (Figure S3b, Supporting Information) were comparable; therefore, NH(3) is almost coplanar to the benzoyl moiety and transoid with respect to NH(2). On this basis, a possible cisoid arrangement of NH(3) and carbonyl function (which would lead the amide proton NH(3) far away from the benzoyl aromatic ring) can be also ruled out in favor of their transoid arrangement. Very low-intensity NH(3)–CH_3_ ROE was detected (Supporting Information, Figure S3a,c), which supported the cisoid arrangement of NH(3) and the thiocarbonyl group; as a matter of fact, their transoid relative positions would bring H_3_ close to the CH–CH_3_ moiety. Finally, the H_7_ proton of the phenolic moiety must be in the proximity of the CH–CH_3_ fragment far away from NH(2), as supported by the ROE patterns produced by perturbation at the H_7_ frequency (Supporting Information, Figure S3d).

**Figure 6:**
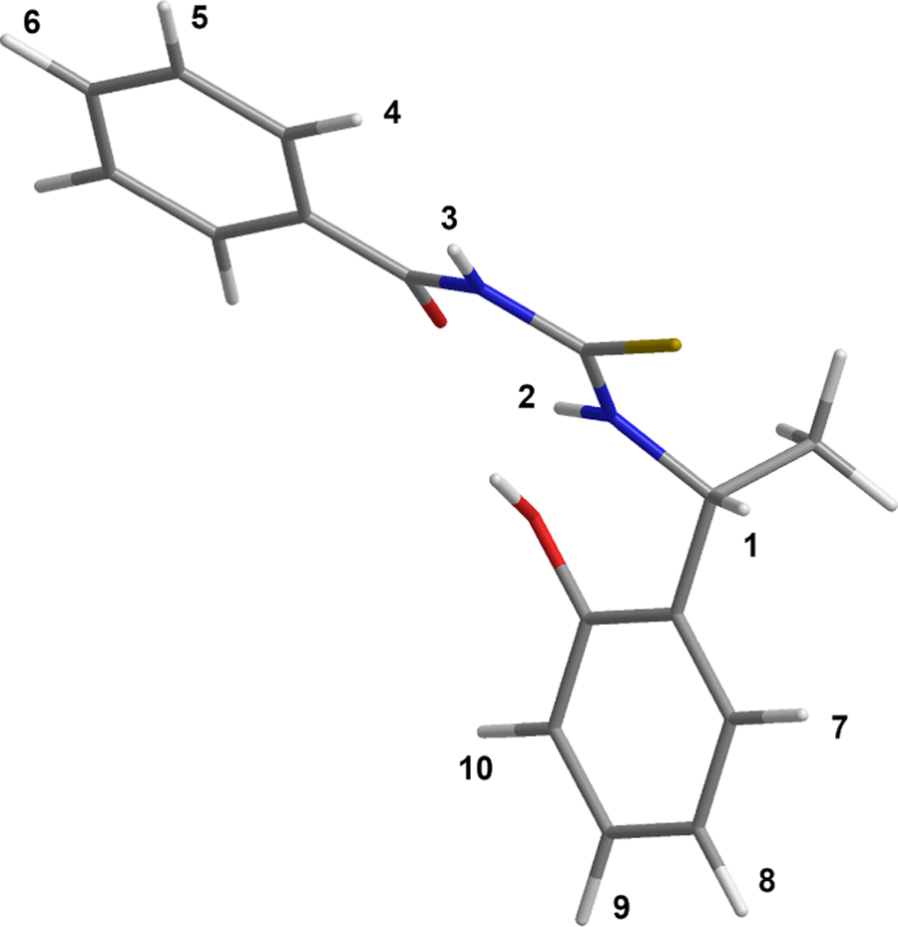
Representation of the three-dimensional (3D) structure of **1-TU** according to ROE data.

Therefore, phenolic OH, NH(2), and the carbonyl moiety are all in proximity, probably due to the formation of an extended pool of acceptor/donor hydrogen bond interactions. In such a way, a flexible pocket-like conformation (Figure 6) is stabilized, inside which not only extended hydrogen bond interactions can be established, responsible for the stabilization of diastereomeric complexes with enantiomeric substrates but also relevant anisotropic effects can be exerted by the two aromatic moieties of the CSA.

Comparison of intermolecular dipolar interactions detected in the equimolar mixtures **1-TU**/(*S*)-**5**/DABCO and **1-TU**/(*R*)-**5**/DABCO allowed to ascertain the nature of the interactions responsible for the stabilization of the two diastereomeric solvates. ROE patterns originated by the methyl and methine protons of **1-TU** were particularly informative: in the mixture containing (*S*)-**5**, the methyl proton of CSA originated only intramolecular dipolar interactions (Supporting Information, Figure S4a), whereas its methine proton (Supporting Information, Figure S4b) produced ROEs both at the amide proton of (*S*)-**5** and at its phenyl moiety. The reverse was found for the mixture containing (*R*)-**5**, with the methine proton of the CSA producing only intramolecular dipolar interactions (Supporting Information, Figure S5b) and its methyl protons showing intermolecular dipolar interactions with the 3,5-dinitrobenzoyl moiety of (*R*)-**5** (Supporting Information, Figure S5a). Accordingly, a reversal of magnitudes of complexation shifts was found for the methine and methyl protons of the CSA in the two mixtures (Table 5). The presence of (*S*)-**5** produced a complexation shift of −0.19 ppm at the methine proton of the CSA and a minor effect at its methyl protons (−0.09 ppm); in the mixture containing (*R*)-**5**, the methyl protons of **1-TU** underwent a greater shift (−0.11 ppm) in comparison with the methine proton (−0.06 ppm).

**Table 5:** Complexation Shifts (Δδ = δ_mixture_ – δ_free_, ppm) of Methyl and Methine Protons of the Two Enantiomers of **5** (30 mM) in the Presence of 1 equiv of DABCO and of **1-TU**

	Δδ	
**1-TU**	(*R*)-**5**/DABCO/**1-TU**	(*S*)-**5**/DABCO/**1-TU**
CH	–0.06	–0.19
CH_3_	–0.11	–0.09
NH(2)	0.06	0.09
NH(3)	0.03	0.03

Protons of the phenolic moiety of **1-TU** were produced through space dipolar interactions with the 3,5-dinitrobenzoyl groups of both enantiomeric substrates (Supporting Information, Figure S6).

Interestingly, different ^15^N complexation shifts of NH(2) and NH(3) groups of **1-TU** were detected in the ^1^H–^15^N HSQC map of the mixture **1-TU**/**5**/DABCO (Supporting Information, Figure S7), −0.8 and +0.5 ppm, respectively, suggesting that NH(2) is more effectively involved in the stabilization of the diastereomeric solvates. The major involvement of NH(2) is also witnessed by the fact that NH(2) underwent greater ^1^H complexation shifts in comparison with NH(3) in both mixtures (Table 5) and the thiocarbonyl group of the CSA underwent remarkably higher ^13^C complexation shifts in comparison with the carbonyl group (−0.289 and −0.075 ppm, respectively, in the mixture containing the racemic phenylglycine derivative).

**Figure 7:**
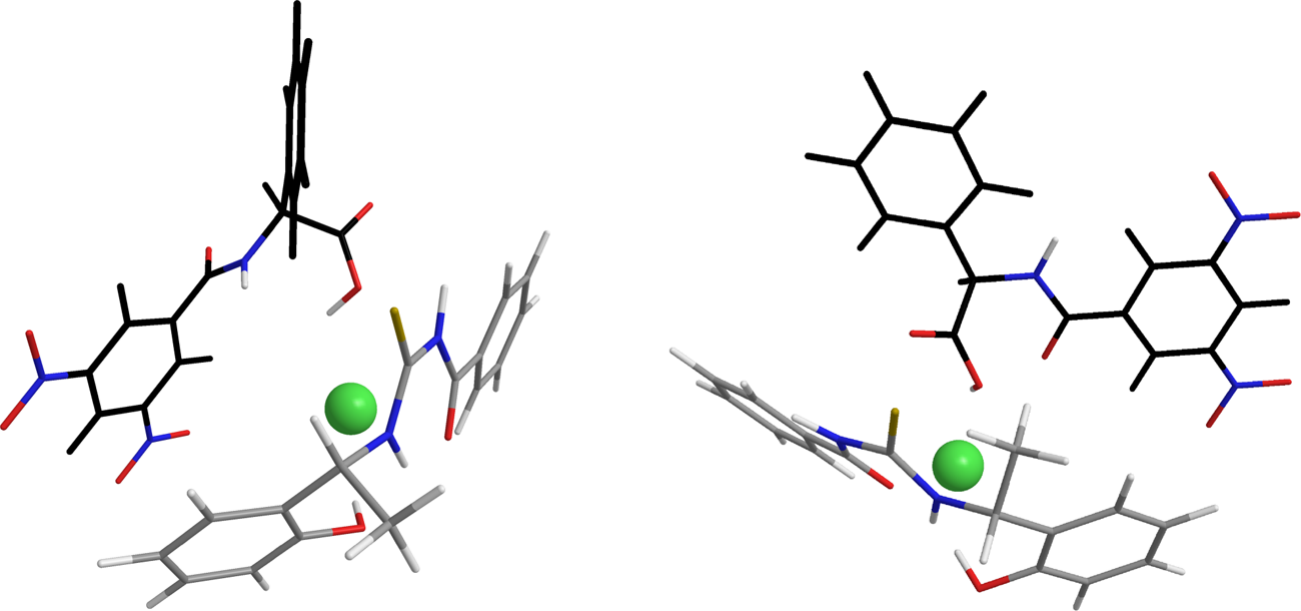
Representation of the interaction model between **1-TU** and (*S*)-**5** (left) or (*R*)-**5** (right) in the presence of DABCO (green sphere) according to NMR data with a black skeleton for **5**.

Finally, DABCO produced multiple intense ROEs at the frequencies of protons of the CSA and of the enantiomeric substrates (Supporting Information, Figure S8). Therefore, it can be concluded that in both diastereomeric solvates, DABCO acts as a bridge between the carboxyl function of the two enantiomers and the pool of hydrogen bond donor/acceptor groups of the CSA and the electron-rich phenolic aromatic moiety of the CSA is involved in π–π interactions with the 3,5-dinitrobenzoyl moiety of both enantiomers. In this way, (*S*)-**5** and (*R*)-**5** face opposite sides of the CSA, pointing at its CH and CH_3_ groups, respectively (Figure 7). Probably, approaching the same less hindered surface of the CSA does not allow a hydrogen bond and π–π interactions to be simultaneously guaranteed.

The active role of DABCO in the stabilization of diastereomeric solvates has been also confirmed by comparison of its diffusion coefficient (*D*), measured by diffusion-ordered spectroscopy (DOSY),^24^ as a pure compound, in the binary mixtures (*R*)-**5**/DABCO and (*S*)-**5**/DABCO and in the ternary mixtures **1-TU**/(*R*)-**5**/DABCO and **1-TU**/(*S*)-**5**/DABCO.

Diffusion coefficients describe the translational diffusion of the molecules in solution and can be correlated to the molecular sizes by means of the Stokes–Einstein equation (eq 2), which strictly holds for spherical molecules
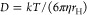
2where *k* is the Boltzmann constant, *T* the absolute temperature, *r*_H_ the hydrodynamic radius, and η the solution viscosity. For quite diluted solutions, viscosity is not affected significantly by the presence of a solute and it can be approximated to the viscosity of the solvent.

In the case of complexation equilibria, the measured diffusion coefficient (*D*_obs_) in fast exchanging conditions represents the weighted average of its values in the bound (*D*_b_) and free (*D*_f_) states (eq 3)
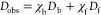
3where χ_b_ and χ_f_ are the molar fractions of bound and free species, respectively.

Any complexation phenomenon, therefore, brings about an increase of the apparent molecular sizes, which in turn causes a decrease of the diffusion coefficient, the magnitude of which depends on the value of the bound molar fraction.

In our case, the diffusion coefficient of pure DABCO was 12.3 × 10^–10^ m^2^ s^–1^ (30 mM in CDCl_3_), which remarkably lowered to 7.3 × 10^–10^ m^2^ s^–1^ in the presence of an equimolar amount of **5**: this value was very similar to that measured for **5** (6.9 × 10^–10^ m^2^ s^–1^), to indicate the formation of a tight ionic pair. DABCO diffusion motion was further affected in the ternary mixture **1-TU**/**5**/DABCO, where its diffusion coefficient was even more lowered to the value of 6.4 × 10^–10^ m^2^ s^–1^, which demonstrated that DABCO simultaneously interacted with the enantiomeric substrates and the CSA, acting as a bridge between them.

## Conclusions

The quantitative derivatization reaction of 2-[(1*R*)-1-aminoethyl]phenol with 1 equiv of benzoyl isothiocyanate affords an efficient CSA for the enantiodiscrimination of amino acid derivatives. Provided a 3,5-dinitrobenzoyl group is present at the amino group of the amino acids, any further derivatization at the carboxyl function is not needed to obtain efficient enantiodifferentiation by the CSA: only an achiral base additive is required to attain solubilization in CDCl_3_. Very high nonequivalences are measured in the presence of **1-TU** up to about 0.2 ppm in the ^1^H NMR spectra and even more in the ^13^C{^1^H} NMR spectra. ^15^N nuclei of enantiomeric substrates can be differentiated too. Enantiodifferentiations remain considerable also in the mixtures containing quite low CSA concentrations (5 mM) with obvious advantages from the economic point of view. The role of DABCO is not restricted to its solubilizing effects on the amino acid derivatives, but rather the base promotes the stabilization of the diastereomeric solvates since it acts as a bridge between the CSA and the enantiomeric substrates for the enhancement of hydrogen bond donor/acceptor propensities of the polar groups of the two counterparts. The phenolic hydroxy group of the CSA plays a fundamental role in two respects: it is endowed with enhanced hydrogen bond donor propensity and makes electron-rich the aromatic ring it is bound to, thus simultaneously favoring π–π interactions with the electron-poor 3,5-dinitrobenzoyl moiety of the amino acid derivatives. This last interaction is so much relevant that, to preserve it, the two enantiomers approach the two different faces of the CSA, the one containing the methine hydrogen at the chiral center in the case of the (*S*)-enantiomer and its methyl group in the case of the (*R*)-enantiomer, from which chiral discrimination originates.

## Experimental Section

### Materials

All commercially available substrates, reagents, and solvents were purchased from Sigma-Aldrich and used without further purification. Tetrahydrofuran (THF) was distilled from sodium. Derivative **10** and derivatives **5**–**9**, **11**–**17** were prepared as described in refs (25, 26), respectively.

### General Methods

^1^H and ^13^C{^1^H} NMR measurements were carried out in a CDCl_3_ solution on a spectrometer operating at 600, 150, and 60.7 MHz for ^1^H, ^13^C, and ^15^N nuclei, respectively. The samples were analyzed in the CDCl_3_ solution, ^1^H and ^13^C chemical shifts are referred to tetramethylsilane (TMS) as the secondary reference standard, ^15^N chemical shifts are referred to nitromethane as the external standard, and the temperature was controlled (25 °C). For all of the 2D NMR spectra, the spectral width used was the minimum required in both dimensions. The gCOSY (gradient COrrelation SpectroscopY) and TOCSY (TOtal Correlation SpectroscopY) maps were recorded using a relaxation delay of 1 s, 256 increments of 4 transients, each with 2 *K*-points. For TOCSY maps, a mixing time of 80 ms was set. The 2D-ROESY (Rotating-frame Overhauser Enhancement SpectroscopY) maps were recorded using a relaxation time of 5 s and a mixing time of 0.5 s; 256 increments of 16 transients of 2 *K*-points each were collected. The 1D-ROESY spectra were recorded using a selective inversion pulse, transients ranging from 256 to 1024, a relaxation delay of 5 s, and a mixing time of 0.5 s. The gHSQC (gradient Heteronuclear Single Quantum Coherence) spectra were recorded, with a relaxation time of 1.2 s, 128–256 increments with 32 transients, each of 2 *K-*points. The gHMBC (gradient Heteronuclear Multiple Bond Correlation) experiments were optimized for a long-range coupling constant of 8 Hz. DOSY (Diffusion-Ordered SpectroscopY) experiments were carried out using a stimulated echo sequence with self-compensating gradient schemes and 64 *K* data points. Typically, *g* was varied in 20 steps (2–32 transients each) and Δ and δ were optimized to obtain an approximately 90–95% decrease in the resonance intensity at the largest gradient amplitude. The baselines of all arrayed spectra were corrected prior to processing the data. After data acquisition, each FID was apodized with 1.0 Hz line broadening and Fourier transformed. The data were processed with the DOSY macro (involving the determination of the resonance heights of all of the signals above a pre-established threshold and the fitting of the decay curve for each resonance to a Gaussian function) to obtain pseudo-two-dimensional spectra with NMR chemical shifts along one axis and calculated diffusion coefficients along the other.

^1^H NMR and ^13^C{^1^H} NMR characterization data, reported below, refer to numbered protons/carbons of chemical structures reported in Figures S9 and S10 (Supporting Information).

### Synthesis of Chiral Auxiliaries **1-TU**, **2-TU**, **3-TU**, and **4-TU**

To a suspension of **1**–**4** (2 mmol) in CH_2_Cl_2_ (20 mL) was added, under a nitrogen atmosphere, benzoyl isothiocyanate (1.1 equiv). The reaction mixture was stirred at room temperature for 24 h. The reaction was monitored by recording ^1^H NMR and the solvent was removed by evaporation under vacuum to afford chemically pure products in a nearly quantitative yield.

**1-TU**: Amber amorphous solid (598 mg, 99.5% yield). ^1^H NMR (600 MHz, CDCl_3_, 25 °C) δ: 1.71 (Me, d, *J* = 6.9 Hz, 3H); 5.81 (H-1, dq, *J* = 8.1 Hz, *J* = 6.9 Hz, 1H); 6.77 (H-11, s, 1H); 6.90 (H-10, d, *J* = 7.9 Hz, 1H); 6.94 (H-8, t, *J* = 7.9 Hz, 1H); 7.19 (H-9, t, *J* = 7.9 Hz, 1H); 7.30 (H-7, d, *J* = 7.9 Hz, 1H); 7.50 (H-5, t, *J* = 7.8 Hz, 2H); 7.61 (H-6, t, *J* = 7.8 Hz, 1H); 7.79 (H-4, d, *J* = 7.8 Hz, 2H); 8.92 (H-3, br s, 1H); 11.27 (H-2, d, *J* = 8.1 Hz, 1H). ^13^C{^1^H} NMR (150 MHz, CDCl_3_, 25 °C) δ: 20.1 (C-Me); 51.3 (C-1); 117.5 (C-10); 121.1 (C-8); 127.4 (C-4, C-15); 127.5 (C-7); 129.2 (C-5, C-9); 131.6 (C-12); 133.7 (C-6); 153.8 (C-16); 166.9 (C-13); 178.4 (C-14). Anal. calcd for C_16_H_16_N_2_SO_2_: C, 63.98; H, 5.37; N, 9.33. Found: C, 63.90; H, 5.38; N, 9.35.

**2-TU**: White solid (621 mg, 99.4% yield). ^1^H NMR (600 MHz, CDCl_3_, 25 °C) δ: 2.28 (H-8, s, 1H); 3.04 (H-9, dd, *J* = 16.6 Hz, *J* = 2.2 Hz, 1H); 3.27 (H-9′, dd, *J* = 16.6 Hz, *J* = 5.3 Hz, 1H); 4.90 (H-7, dt, *J* = 5.3 Hz, *J* = 2.2 Hz, 1H); 5.93 (H-1, dd, *J* = 7.8 Hz, *J* = 5.3 Hz, 1H); 7.26 (H-12, m, 1H); 7.29 (H-11, m, 1H; H-10, d, *J* = 6.8 Hz, 1H); 7.46 (H-13, d, *J* = 7.3 Hz, 1H); 7.50 (H-5, t, *J* = 7.4 Hz, 2H); 7.62 (H-6, t, *J* = 7.4 Hz, 1H); 7.84 (H-4, d, *J* = 7.4 Hz, 2H); 9.14 (H-3, s, 1H); 11.20 (H-2, d, *J* = 7.8 Hz, 1H). ^13^C{^1^H} NMR (150 MHz, CDCl_3_, 25 °C) δ: 39.8 (C-9); 63.8 (C-1); 73.5 (C-7); 124.9 (C-13); 125.5 (C-10); 127.4 (C-12); 127.5 (C-4); 128.7 (C-11); 129.1 (C-5); 131.7 (C-14); 133.6 (C-6); 139.2 (C-18); 139.9 (C-17); 166.6 (C-15); 180.6 (C-16). Anal. calcd for C_17_H_16_N_2_SO_2_: C, 65.36; H, 5.16; N, 8.97. Found: C, 65.29; H, 5.15; N, 8.95.

**3-TU**: Brownish solid (623 mg, 99.6% yield). ^1^H NMR (600 MHz, CDCl_3_, 25 °C) δ: 3.03 (H-9, dd, *J* = 16.2 Hz, *J* = 6.8 Hz, 1H); 3.43 (H-9′, dd, *J* = 16.2 Hz, *J* = 7.8 Hz, 1H); 4.05 (H-8, s, 1H); 4.71 (H-7, ddd, *J* = 7.8 Hz, *J* = 6.8 Hz; *J* = 5.7 Hz, 1H); 5.73 (H-1, t, *J* = 5.7 Hz, 1H); 7.26 (H-10, d, *J* = 7.1 Hz, 1H); 7.29 (H-12, t, *J* = 7.5 Hz, 1H); 7.31 (H-11, dd, *J* = 7.5 Hz, *J* = 7.1 Hz, 1H); 7.35 (H-13, d, *J* = 7.5 Hz, 1H); 7.53 (H-5, t, *J* = 7.8 Hz, 2H); 7.64 (H-6, t, *J* = 7.8 Hz, 1H); 7.85 (H-4, d, *J* = 7.8 Hz, 2H); 9.14 (H-3, s, 1H); 11.11 (H-2, d, *J* = 5.7 Hz, 1H). ^13^C{^1^H} NMR (150 MHz, CDCl_3_, 25 °C) δ: 39.3 (C-9); 68.9 (C-1); 81.0 (C-7); 123.8 (C-13); 125.3 (C-10); 127.5 (C-4); 127.6 (C-12); 129.1 (C-11); 129.2 (C-5); 131.5 (C-14); 133.8 (C-6); 138.3 (C-18); 140.8 (C-17); 167.0 (C-15); 181.0 (C-16). Anal. calcd for C_17_H_16_N_2_SO_2_: C, 65.36; H, 5.16; N, 8.97. Found: C, 65.43; H, 5.16; N, 8.95.

**4-TU**: Yellow gum (565 mg, 99.3% yield). ^1^H NMR (600 MHz, CDCl_3_, 25 °C) δ: 1.66 (Me, d, *J* =7.1 Hz, 3H); 5.62 (H-1, dq, *J* = 7.4 Hz, *J* = 7.1 Hz, 1H); 7.29 (H-9, t, *J* = 7.4 Hz, 1H); 7.37 (H-8, t, *J* = 7.4 Hz, 2H); 7.40 (H-7, d, *J* = 7.4 Hz, 2H); 7.51 (H-5, t, *J* = 7.5 Hz, 2H); 7.62 (H-6, t, *J* = 7.8 Hz, 1H); 7.82 (H-4, d, *J* = 7.8 Hz, 2H); 8.96 (H-3, s, 1H); 11.11 (H-2, d, *J* = 7.4 Hz, 1H). ^13^C{^1^H} NMR (150 MHz, CDCl_3_, 25 °C) δ: 21.6 (C-Me); 55.2 (C-1); 126.3 (C-7); 127.4 (C-4); 127.7 (C-9); 128.8 (C-8); 129.1 (C-5); 131.8 (C-10); 133.6 (C-6); 141.6 (C-13); 166.8 (C-11); 178.8 (C-12). Anal. calcd for C_16_H_16_N_2_SO: C, 67.58; H, 5.67; N, 9.85. Found: C, 67.63; H, 5.66; N, 9.87.

### Synthesis of *N-*3,5-Dinitrobenzoyl Amino Acids **5**–**9** and of *N-*3,5-Dimethoxybenzoylalanine (**11**)

A solution of the appropriate amino acid (2 mmol), propylene oxide (6 mmol), and *N-*3,5-dinitrobenzoyl chloride (or *N-*3,5-dimethoxybenzoyl chloride) (2 mmol) in anhydrous THF (30 mL) was stirred, under a nitrogen atmosphere, overnight at room temperature. The residue, obtained by solvent evaporation under reduced pressure, was dissolved in ethyl acetate and treated with a solution of HCl (10%), a saturated solution of NaCl, and dried over anhydrous Na_2_SO_4_. The crude product was suspended in petroleum ether/ethanol (5:1, 10 mL) at 0 °C under stirring for 10–15 min. The product was filtered and dried under vacuum. ^1^H NMR (600 MHz, 25 °C, 30 mM) spectra of **5**–**9**, and **11**, reported below, were recorded in a CDCl_3_ solution in the presence of 1 equiv of DABCO.

**5**: White crystalline solid; 78% yield (539 mg). ^1^H NMR δ (ppm): 5.47 (H-1, d, *J* = 6.0 Hz, 1H); 7.24 (H-4, t, *J* = 7.6 Hz, 1H); 7.31 (H-3, t, *J* = 7.6 Hz, 2H); 7.49 (H-2, d, *J* = 7.6 Hz, 2H); 8.35 (H-5, d, *J* = 6.0 Hz, 1H); 9.01 (H-6, d, *J* = 2.1, 2H); 9.11 (H-7, t, *J* = 2.1, 1H).

**6**: Mustard yellow crystalline solid; 75% yield (488 mg). ^1^H NMR δ (ppm): 0.95 (H-4, d, *J* = 6.5 Hz, 3H); 0.98 (H-4′, d, *J* = 6.5 Hz, 3H); 1.81 (H-3, H-2, and H-2′, m, 3H); 4.63 (H-1, dt, *J* = 7.8 Hz, *J* = 6.5 Hz, 1H); 7.98 (H-5, d, *J* = 7.8 Hz,1H); 8.98 (H-6, d, *J* = 2.0 Hz, 2H); 9.09 (H-7, t, *J* = 2.0 Hz, 1H).

**7**: Pale brown amorphous solid; 72% yield (408 mg). ^1^H NMR δ (ppm): 1.54 (H-2, d, *J* = 6.7 Hz, 3H); 4.50 (H-1, quint, *J* = 6.7 Hz, 1H); 7.80 (H-3, d; *J* = 6.7 Hz, 1H); 9.00 (H-4, d, *J* = 1.9 Hz, 2H); 9.18 (H-5, t, *J* = 1.9 Hz, 1H).

**8**: White amorphous solid; 78% yield (486 mg). ^1^H NMR δ (ppm): 0.99 (H-3, d, *J* = 6.8 Hz, 3H); 1.12 (H-3′, d, *J* = 6.8 Hz, 3H); 2.34 (H-2, m, 1H); 4.59 (H-1, dd, *J* = 7.9 Hz, *J* = 4.3 Hz, 1H); 7.54 (H-4, d, *J* = 7.9 Hz, 1H); 9.00 (H-5, d, *J* = 2.0 Hz, 2H); 9.10 (H-6, t, *J* = 2.0, 1H).

**9**: White crystalline solid; 77% yield (553 mg). ^1^H NMR δ (ppm): 3.27 (H-2, dd, *J* = 13.5 Hz, *J* = 5.3 Hz, 1H); 3.41 (H-2′, dd, *J* = 13.5 Hz, *J* = 5.3 Hz, 1H); 4.79 (H-1, m, 1H); 7.16–7.24 (H-3, H-4, and H-5, m, 5H); 7.70 (H-6, d, *J* = 6.7 Hz, 1H); 8.87 (H-7, d, *J* = 2.0, 2H); 9.27 (H-8, t, *J* = 2.0, 1H).

**11**: Pale brown crystalline solid; 70% yield (355 mg). ^1^H NMR δ (ppm): 1.51 (H-2, d, *J* = 7.2 Hz, 3H); 3.81 (H-6, s, 6H); 4.77 (H-1, quint, *J* = 7.2 Hz, 1H), 6.66 (H-5, t, *J* = 2.3 Hz, 1H), 6.68 (H-4, d, *J* = 2.3, 2H), 6.91 (H-3, d, *J* = 7.2 Hz, 1H).

### Synthesis of *N*-Trifluoroacetylvaline (**10**)

To a solution of valine (11 mmol), triethylamine (11 mmol) in MeOH (10 mL) and ethyl trifluoroacetate (14.3 mmol) were added and the solution was stirred for 24 h. After solvent evaporation, the solid was purified by treating with water and HCl. The organic phase was extracted in ethyl acetate, washed with a saturated solution of NaCl, and dried with anhydrous Mg_2_SO_4_. By removing the solvent by evaporation under reduced pressure, racemate **10** was obtained as a white crystalline solid (1.990 g, 85% yield). ^1^H NMR (600 MHz, CDCl_3_, 25 °C) in the presence of 1 equiv of DABCO, δ (ppm): 0.94 (H-3, d, *J* = 6.7 Hz, 3H); 0.95 (H-3′, d, *J* = 6.7 Hz, 3H); 2.27 (H-2, m, 1H), 4.29 (H-1, dd, *J* = 8.7 Hz, *J* = 4.5 Hz, 1H), 7.27 (H-4, d, *J* = 8.7 Hz, 1H).

### Synthesis of *N-*3,5-Dinitrobenzoyl Derivatives of Amino Acid Methyl Esters **12**–**14**

A solution of **5**, **7** or **8** (3 mmol) in anhydrous MeOH (30 mL) saturated with HCl gas was refluxed for 1 h. The crude product, obtained by solvent evaporation, was dissolved in CH_2_Cl_2_, and washed with a saturated NaHCO_3_ solution, H_2_O, and dried over Na_2_SO_4_. **12**–**14** were obtained chemically pure.

**12**: White crystalline solid; 78% yield (842 mg). ^1^H NMR (600 MHz, CDCl_3_, 25 °C), δ (ppm): 3.81 (H-8, s, 3H); 5.78 (H-1, d, *J* = 6.8, 1H); 7.35–7.46 (H-2, H-3, and H-4, m, 5H), 7.50 (H-5, d, *J* = 6.8 Hz, 1H); 8.98 (H-6, d, *J* = 1.9 Hz, 2H); 9.16 (H-7, t, *J* = 1.9 Hz, 1H).

**13**: Pale brown amorphous solid; 80% yield (781 mg). ^1^H NMR (600 MHz, CDCl_3_, 25 °C), δ (ppm): 1.03 (H-3, d, *J* = 6.5 Hz, 3H); 1.04 (H-3′, d, *J* = 6.5, 3H); 2.30 (H-2, m, 1H); 3.81 (H-7, s, 3H); 4.81 (H-1, dd, *J* = 8.5 Hz, *J* = 4.8 Hz, 1H); 6.84 (H-4, d, *J* = 8.5 Hz, 1H); 8.96 (H-5, d, *J* = 2.2 Hz, 2H); 9.19 (H-6, t, *J* = 2.2 Hz, 1H).

**14**: White crystalline solid; 80% yield (713 mg). ^1^H NMR (600 MHz, CDCl_3_, 25 °C), δ (ppm): 1.56 (H-2, d, *J* = 7.3 Hz, 3H); 3.81 (H-6, s, 3H); 4.81 (H-1, m, 1H); 7.26 (H-3, d, *J* = 6.1 Hz, 1H); 8.93 (H-4, d, *J* = 2.0 Hz, 2H); 9.14 (H-5, t, *J* = 2.0 Hz, 1H).

### Synthesis of *N-*3,5-Dinitrobenzoyl Derivatives of Amino Acid Alkylamides **15** and **16**

To a mixture of **8** or **5** (16 mmol) and 2-ethoxy-1-ethoxycarbonyl-1,2-dihydroquinine (16 mmol) in anhydrous THF (120 mL), stirred under a nitrogen atmosphere at room temperature for 3 h, was added the appropriate amine (8.03 mmol), and stirred at room temperature for a further 15 h. After solvent evaporation, the crude products, **15** and **16**, were purified by recrystallization from THF/hexane.

**15**: White crystalline solid; 45% yield (1.527 g). ^1^H NMR (600 MHz, CDCl_3_, 25 °C), δ (ppm): 0.86 (H-15, t, *J* = 6.8 Hz, 3H); 0.96 (H-3, d, *J* = 6.8 Hz, 3H); 1.02 (H-3′, d, *J* = 6.8 Hz, 3H); 1.20–1.35 (H-10–H14, m, 10H); 1.53 (H-9, m, 2H); 2.14 (H-2, m, 1H); 3.27 (H-8, m, 1H); 3.34 (H-8′, m, 1H); 4.35 (H-1, t, *J* = 8.0 Hz, 1H); 6.18 (H-7, t, *J* = 5.6 Hz, 1H); 8.20 (H-4, d, *J* = 8.0 Hz, 1H); 9.07 (H-5, d, *J* = 2.2 Hz, 2H); 9.15 (H-6, t, *J* = 2.2 Hz, 1H).

**16**: Pale brown crystalline solid; 30% yield (1.100 g). ^1^H NMR (600 MHz, CDCl_3_, 25 °C), δ (ppm): 0.86 (H-16, t, *J* = 7.1 Hz, 3H); 1.15–1.30 (H-11–H15, m, 10H); 1.45 (H-10, m, 2H); 3.27 (H-9, m, 2H); 5.70 (H-1, d, *J* = 6.6 Hz, 1H); 5.83 (H-8, t, *J* = 5.8 Hz, 1H); 7.31 (H-4, t, *J* = 7.5 Hz, 1H); 7.35 (H-3, t, *J* = 7.5 Hz, 2H); 7.47 (H-2, d, *J* = 7.5 Hz, 2H); 8.50 (H5, d, *J* = 6.6 Hz, 1H); 8.97 (H-6, d, *J* = 2.1 Hz, 2H); 9.11 (H-7, t, *J* = 2.1 Hz, 1H).

### Synthesis of *N*-(3,5-Dinitrobenzoyl)-1-phenylethanamine (**17**)

A solution of 3,5-dinitrobenzoyl chloride (8.3 mmol) in THF was added dropwise at 0 °C to a solution of 1-phenylethanamine (8.3 mmol) and triethylamine (9.0 mmol) in anhydrous THF (50 mL). The mixture was stirred at room temperature for 16 h. The reaction was quenched by adding H_2_O. The solvent was removed under reduced pressure and the residue was dissolved in CH_2_Cl_2_; the organic layer was washed with HCl (10%), Na_2_CO_3_ (10%), H_2_O, and dried over anhydrous Na_2_SO_4_. The evaporation of the solvent under reduced pressure afforded (2.250 g, 86% yield) a white crystalline solid, **17**. ^1^H NMR (600 MHz, CDCl_3_, 25 °C) δ: 1.68 (H-2, d, *J* = 6.9 Hz, 3H); 5.36 (H-1, m, 1H); 6.51 (H-6, d, *J* = 7.3 Hz, 1H); 7.30–7.43 (H-3, H-4, and H-5, m, 5H); 8.92 (H-7, d, *J* = 2.0 Hz, 2H); 9.15 (H-8, t, *J* = 2.0 Hz, 1H).

## Supporting Information Available

Role of the base in enantiodiscrimination (Figure S1); nonequivalences in the presence of **1-TU**, **2-TU**, and **4-TU** (Table S1); complexation shifts (Table S2); ^13^C nonequivalences in the presence of **1-TU** (Table S3); nonlinear fittings of dilution data (Figure S2); 1D ROESY spectra (Figures S3–S6 and S8); ^1^H–^15^N HSQC maps (Figure S7); CSA structures with proton and carbon numbering (Figure S9); structures of substrates **5**–**17** with proton numbering (Figure S10); ^1^H NMR (600 MHz, CDCl_3_, 25 °C) and ^13^C{^1^H} NMR (150 MHz, CDCl_3_, 25 °C) spectra of CSAs (Figures S11–S18); and ^1^H NMR (600 MHz, CDCl_3_, 25 °C) spectra of **5**–**17** (Figures S19–S31) (PDF)
