# Supplementary material for: Thiourea Derivative of 2-[(1R)-1-Aminoethyl]phenol: A Flexible Pocket-like Chiral Solvating Agent (CSA) for the Enantiodifferentiation of Amino Acid Derivatives by NMR Spectroscopy
Source: J Org Chem. 2020 Mar 19;85(8):5342–50. doi: 10.1021/acs.joc.0c00027 (PMC7997569; doi:10.1021/acs.joc.0c00027)
Supplement: Supplementary file 1 — jo0c00027_si_001.pdf [file jo0c00027_si_001.pdf]

Thiourea derivative of 2-[(1*R*)-1-aminoethyl]phenol: a flexible pocket like chiral solvating agent (CSA) for the enantiodifferentiation of amino acid derivatives by NMR spectroscopy

Alessandra Recchimurzo, Cosimo Micheletti, Gloria Uccello-Barretta, Federica Balzano*

Department of Chemistry and Industrial Chemistry, University of Pisa, via Moruzzi 13, 56124 Pisa, Italy.

*federica.balzano@unipi.it

Supporting Information

Table of Contents

Role of the base on enantiodiscrimination (Figure S1) S2
Nonequivalences in presence of **1-TU**, **2-TU**, **4-TU** (Table S1) S2
^{1}H Complexation shifts (Table S2) S3
^{13}C Nonequivalences in the presence of **1-TU** (Table S3) S3
Non-linear fitting of dilution data (Figure S2) S4
1D ROESY spectra (Figures S3-S6) S4
^{1}H-^{15}N HSQC maps (Figure S7) S6
1D ROESY spectra (Figure S8) S7
CSAs structures with protons and carbons numbering (Figure S9) S7
Structures of substrates **5**-**17** with protons numbering (Figure S10) S8
^{1}H NMR (600 MHz, $CDCl_3$, 25 °C) and $^{13}C\{^{1}H\}$ NMR (150 MHz, $CDCl_3$, 25 °C) spectra of CSAs (Figures S11-S18) S9
^{1}H NMR (600 MHz, $CDCl_3$, 25 °C) spectra of **5**-**17** (Figures S19-S31) S17

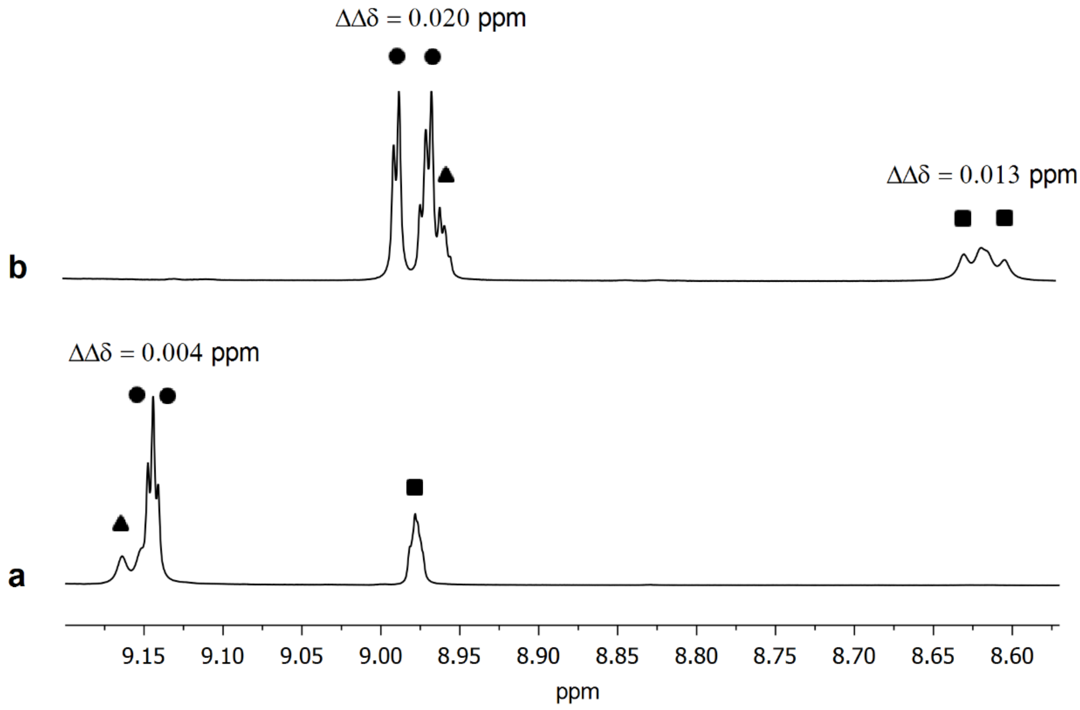

Figure S1. ^{1}H NMR (600 MHz, $CDCl_3$/DMSO-d_6 94:6 (v/v), 25 °C) spectral regions corresponding to 3,5-dinitrobenzoyl (● = ortho, ▲ = para) and NH (■) protons of **5** (15 mM) in **5**/**1-TU** (1:2) mixture (a) and in **5**/**1-TU**/DABCO (1:2:1) mixture (b).

Table S1. ^{1}H NMR (600 MHz, $CDCl_3$, 25 °C) nonequivalences (ppm) of substrates **5**, **6**, **12**, **16** (30 mM) in presence of **1-TU**, **2-TU**, **4-TU** (30 mM).

	**1-TU**				**2-TU**				**4-TU**			
	oDNB	pDNB	NH	CH	oDNB	pDNB	NH	CH	oDNB	pDNB	NH	CH
**5**	0.129	0.079	0.010	0.034	0.004	0.028	0.013	0.006	-	-	0.005	0.005
**6**	0.123	0.076	0.014	nd[a]	0.004	0.054	0.055	0.006	0.014	0.015	-	0.009
**12**	0.071	0.046	0.076	nd[a]	0.002	nd[a]	0.032	0.006	0.001	0.003	nd[a]	-
**16**	0.127	0.071	0.011	nd[a]	0.010	0.034	0.005	0.004	0.004	0.003	-	-

[a]nd = not determined

Table S2. ^{1}H NMR (600 MHz, $CDCl_3$, 25 °C) complexation shifts ($\Delta\delta=\delta_{mixture}-\delta_{free}$, ppm) of (*R*,*S*)-**5** (30 mM) in mixtures **5**/**1-TU**/DABCO (1:1:1), **5**/**2-TU**/DABCO (1:1:1) and **5**/**3-TU**/DABCO (1:1:1).

	$\Delta\delta$		
	**1-TU**	**2-TU**	**3-TU**
**p-DNB**	-0.019	-0.018	-0.003
	-0.105	-0.046	
**o-DNB**	-0.148	-0.009	0.010
	-0.007	-0.005	
**NH**	+0.026	-0.017	-0.087
	+0.003	-0.030	
**CH**	+0.025	+0.003	0.009
	+0.065	+0.009	

Table S3. ^{13}C NMR (150 MHz, $CDCl_3$, 25 °C) nonequivalences (ppm) of **5**, **6**, **12**, **16** (30 mM) in the presence of DABCO (30 mM) and of **1-TU** (30 mM)

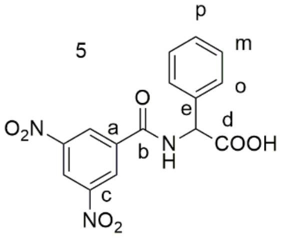

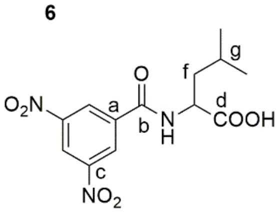

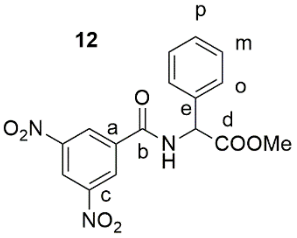

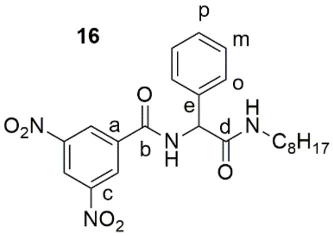

	5	**6**	**12**	**16**
CH	0.236	0.130	0.122	0.228
C^{pDNB}	0.091	0.091	0.053	0.074
C^{oDNB}	0.137	0.038	nd*	0.114
C^{a}	0.196	0.206	0.153	0.207
C^{b}	0.241	0.305	0.137	0.236
C^{c}	0.219	0.252	0.138	0.206
C^{d}	0.313	0.328	0.237	0.227
C^{e}	0.175		0.137	0.251

*nd=not determined

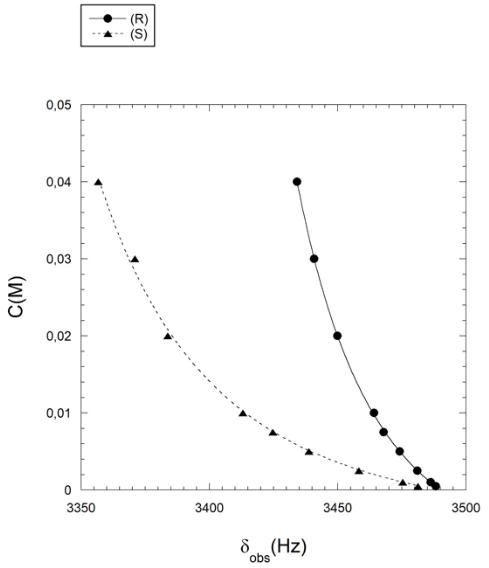

Figure S2. Non-linear fitting of dilution data of (*S*)-**5**/**1-TU**/DABCO and (*R*)-**5**/**1-TU**/DABCO mixtures (1:1:1) based on CH protons of **1-TU**.

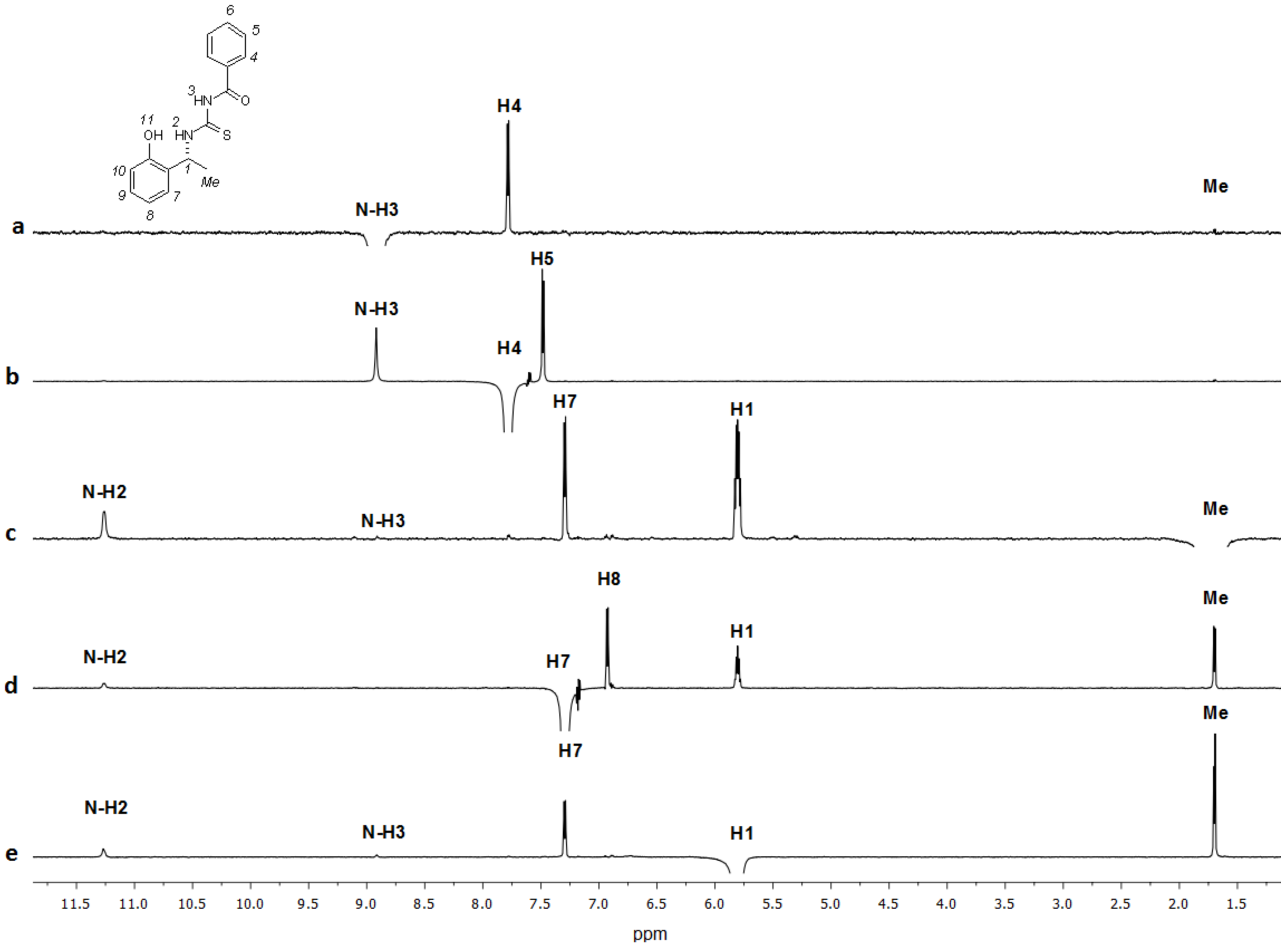

Figure S3. 1D ROESY (600 MHz, $CDCl_3$, 25 °C) spectra of **1-TU** (30 mM) corresponding to the selective perturbation of N-H3 (a), H-4 (b), methyl (c) and H-7 (d) and methine CH-1 (e) protons.

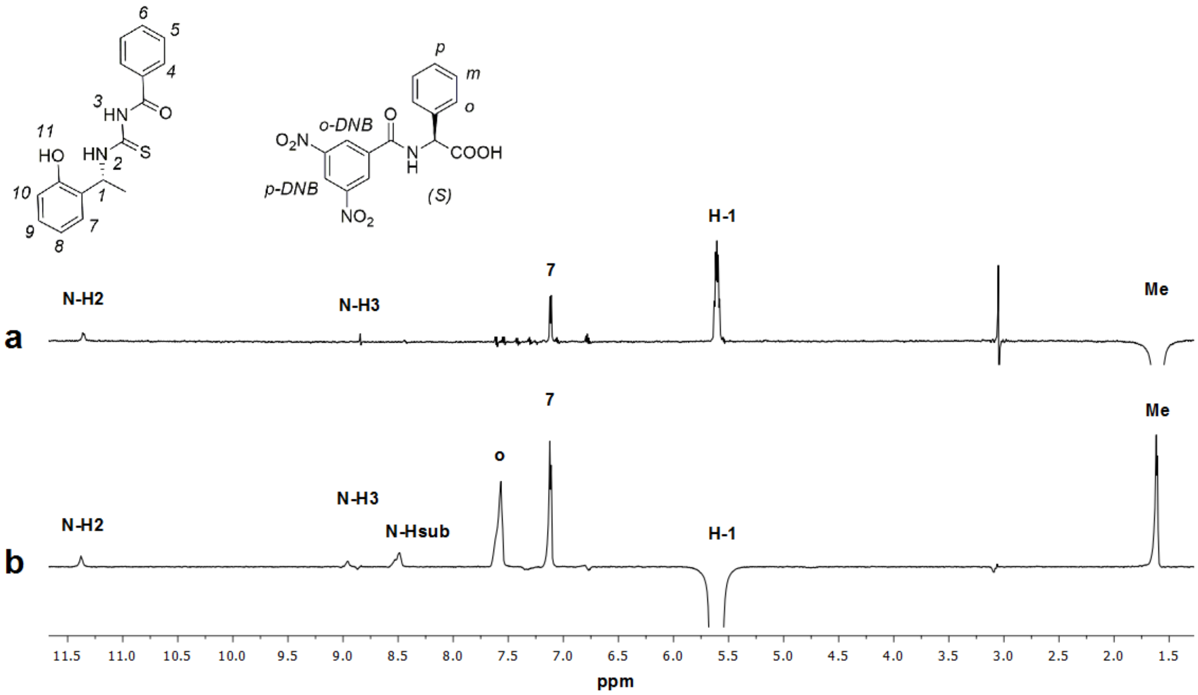

Figure S4. 1D ROESY (600 MHz, $CDCl_3$, 25 °C) spectra corresponding to the selective perturbation of methyl (a) and methine (b) protons of **1-TU** (30 mM) in the mixture (*S*)-**5**/**1-TU**/DABCO (1:1:1).

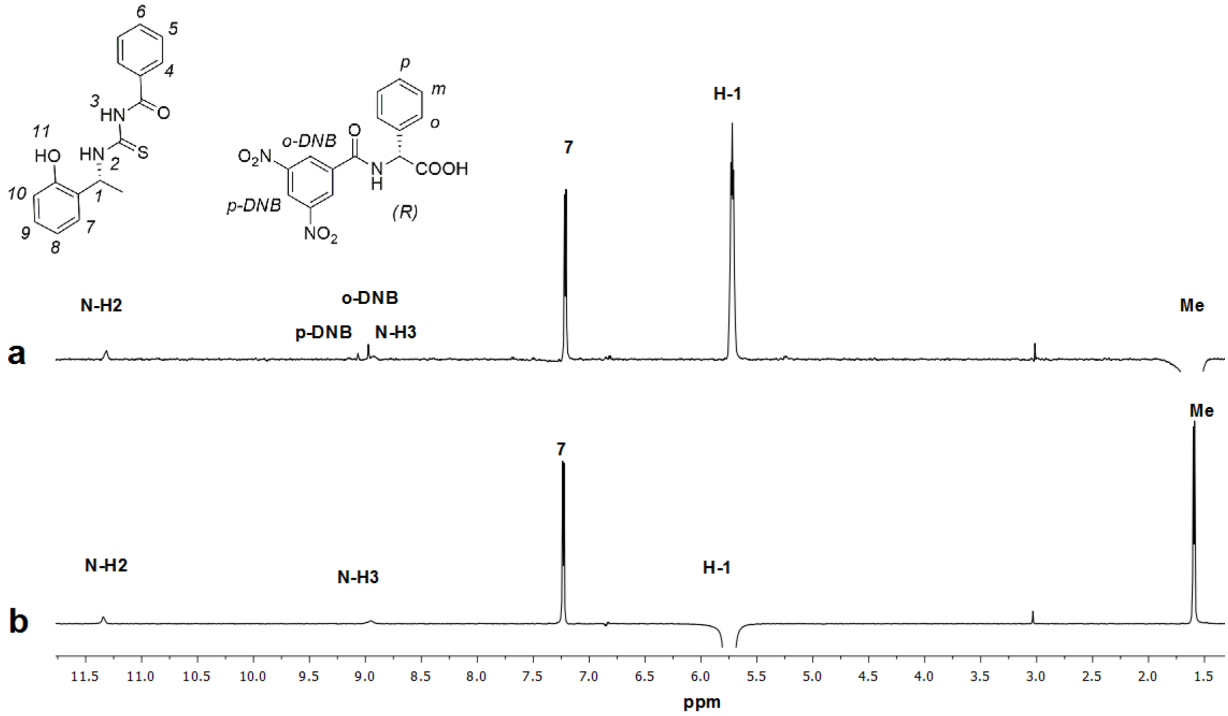

Figure S5. 1D ROESY (600 MHz, $CDCl_3$, 25 °C) spectra corresponding to the selective perturbation of methyl (a) and methine (b) protons of **1-TU** in the mixture (*R*)-**5**/**1-TU**/DABCO (1:1:1).

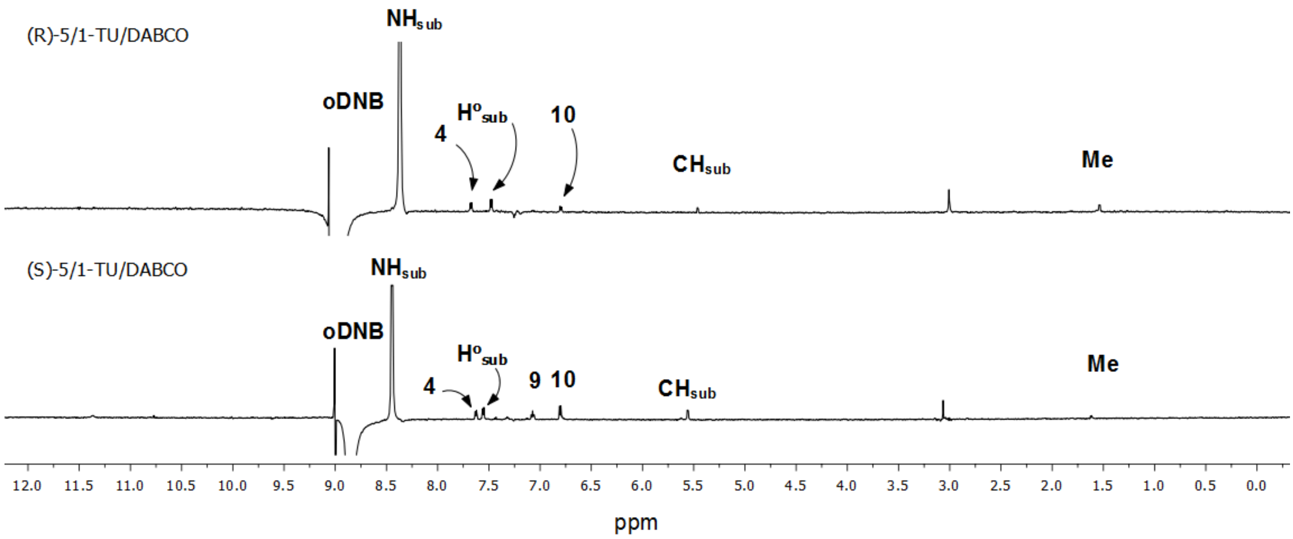

Figure S6. 1D ROESY (600 MHz, $CDCl_3$, 25 °C) spectra corresponding to the selective perturbation of o-DNB protons of **5** (30 mM) in (*R*)-**5**/**1-TU**/DABCO and (*S*)-**5**/**1-TU**/DABCO mixtures (1:1:1).

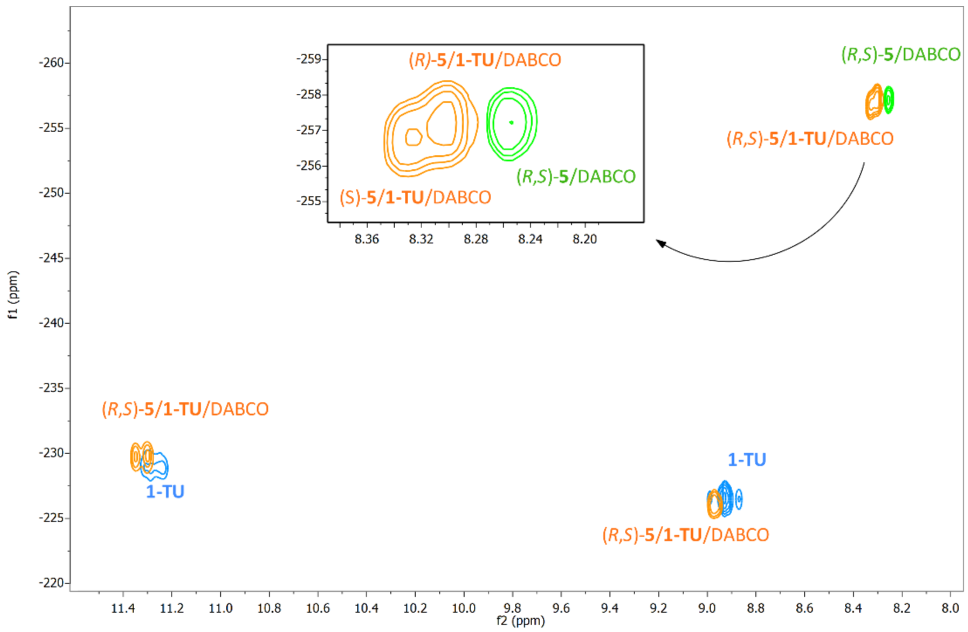

Figure S7. ^{1}H-^{15}N HSQC (600 MHz, $CDCl_3$, 25 °C) maps: spectral regions corresponding to NH protons of (*R*,*S*)-**5** (30 mM) and **1-TU** (30 mM) in **5**/DABCO (1:1) (green), **1-TU** (light blue), and in equimolar **5**/**1-TU**/DABCO mixture (orange).

^{15}N resonances of **5** were affected by the presence of **1-TU**, since doublings of NH resonances of the two enantiomers with respect to **5**/DABCO mixture were detected in the ^{1}H-^{15}N HSQC map of the mixture **1-TU**/**5**/DABCO (Figure S7). In particular, NH group of **5**, which produced in the presence of one equivalent of DABCO a ^{15}N resonance at -257.2 ppm, was at -257.2 ppm and -256.8 ppm for (*R*)-**5** and (*S*)-**5**, respectively, in the presence of **1-TU**, with a nonequivalence of 0.4 ppm.

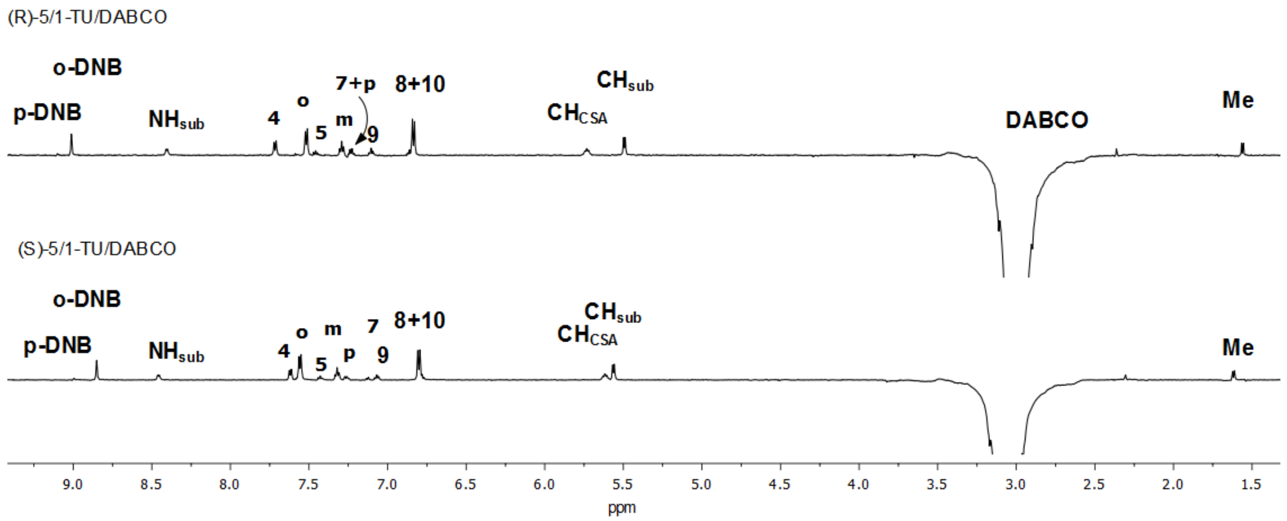

Figure S8. 1D ROESY (600 MHz, $CDCl_3$, 25 °C) spectra corresponding to the selective perturbation of DABCO (30 mM) protons in (*R*)-**5**/**1-TU**/DABCO and (*S*)-**5**/**1-TU**/DABCO mixtures (1:1:1).

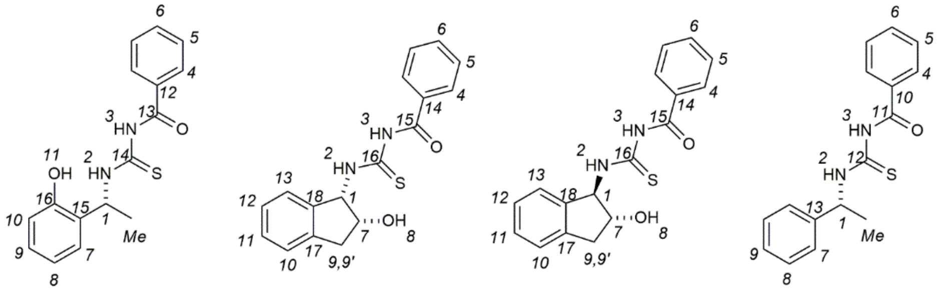

1-TU **2-TU** **3-TU** **4-TU**

Figure S9. CSA structures with protons and carbons numbering.

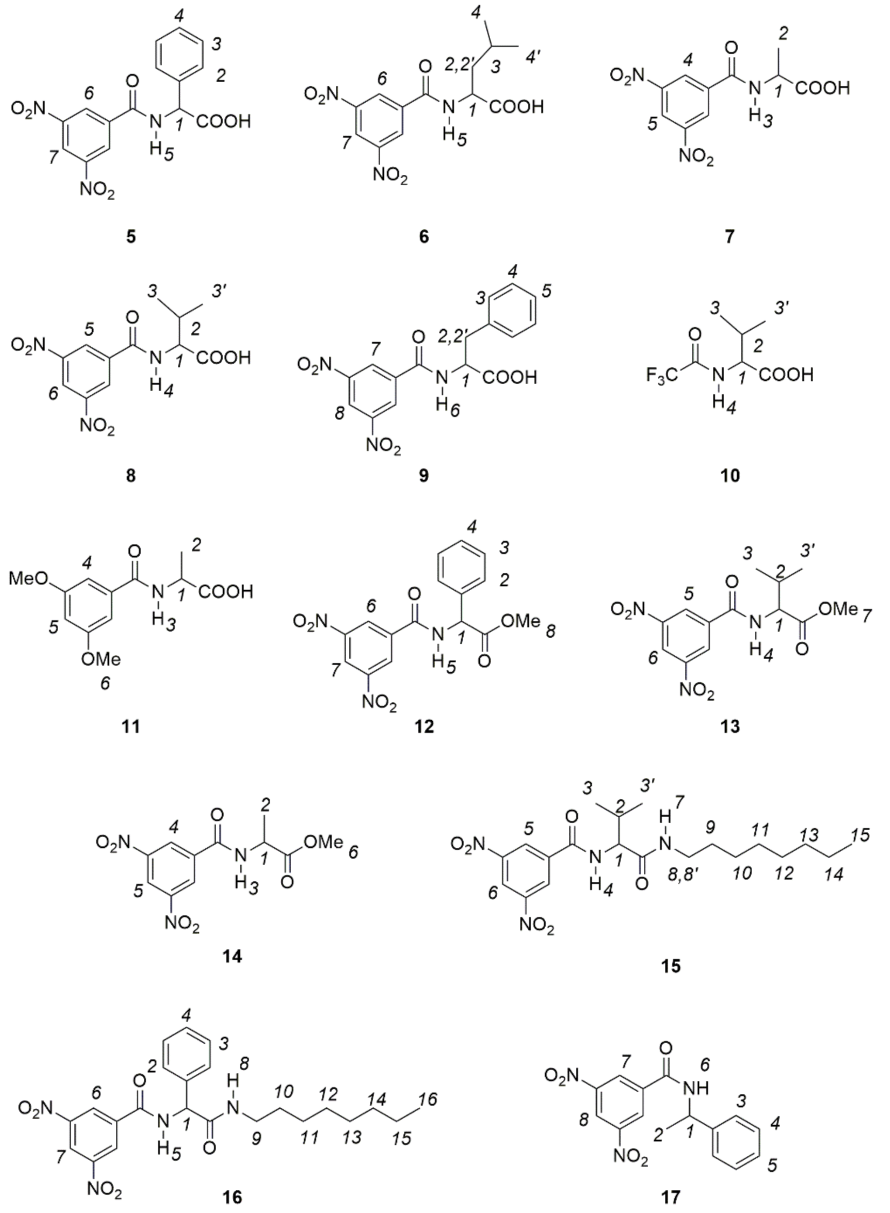

Figure S10. Structures of substrates **5**-**17** with protons numbering.

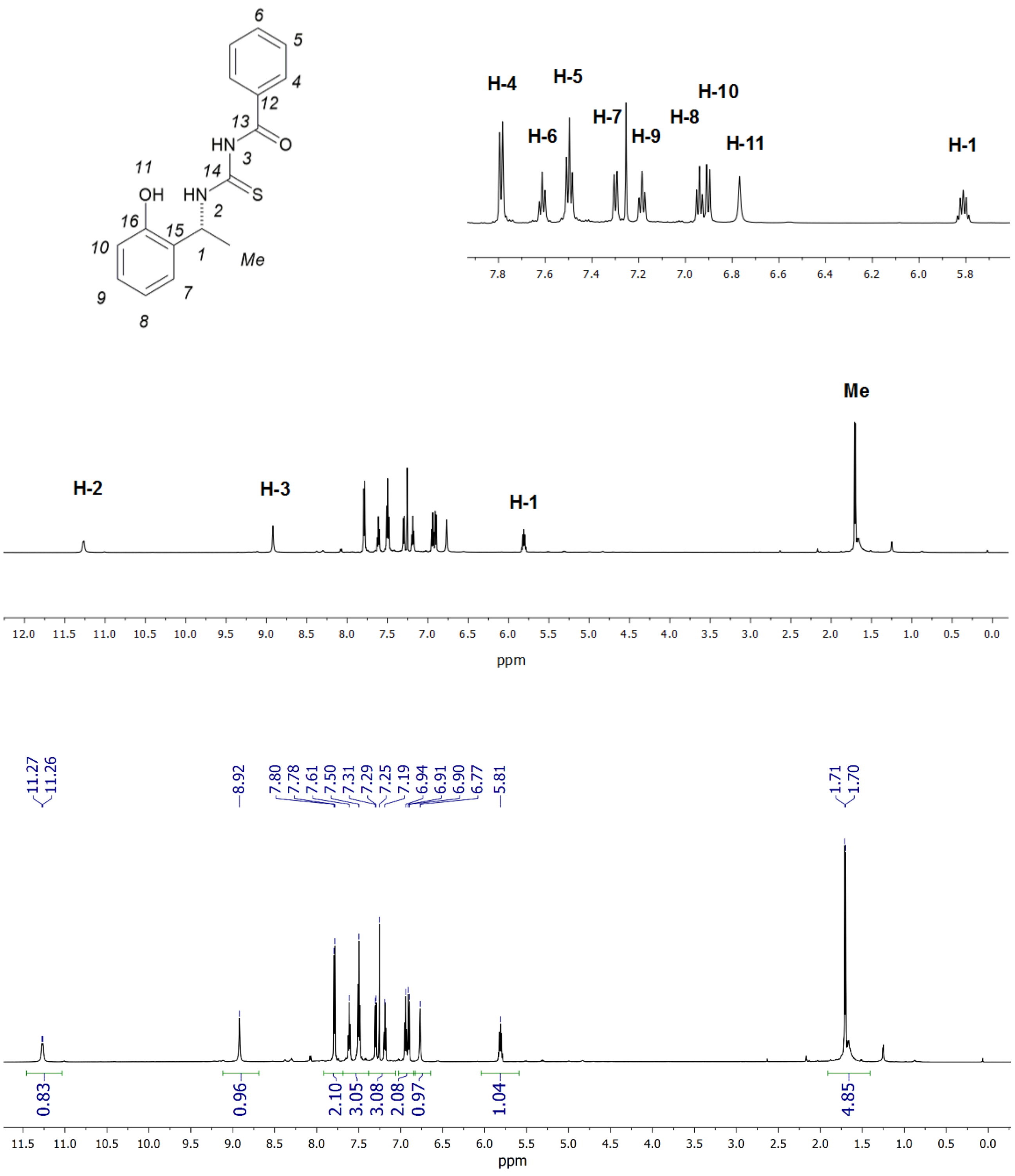

Figure S11. ^{1}H NMR (600 MHz, $CDCl_3$, 25 °C) spectrum of **1-TU**.

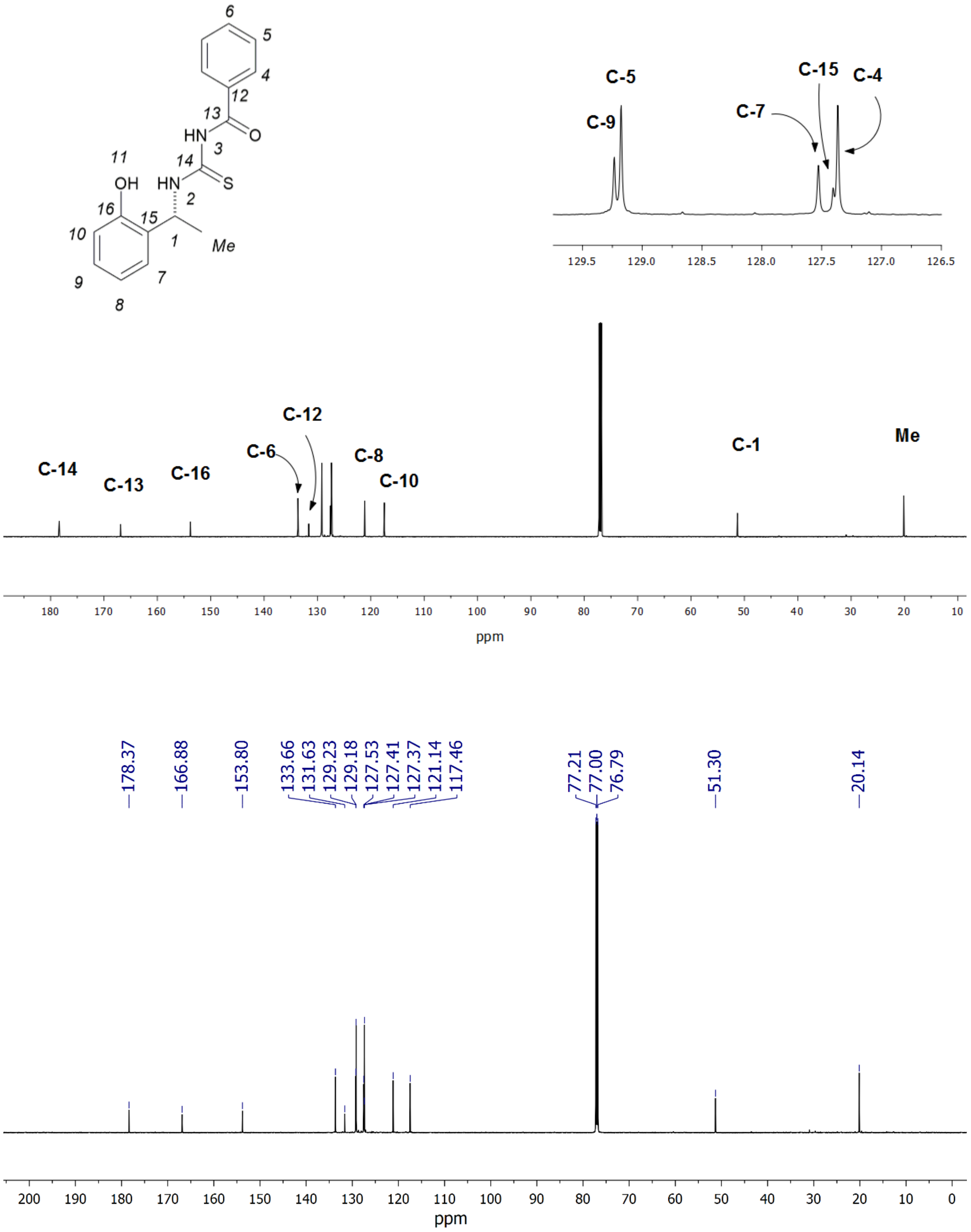

Figure S12. $^{13}C\{^{1}H\}$ NMR (150 MHz, $CDCl_3$, 25 °C) spectrum of **1-TU**.

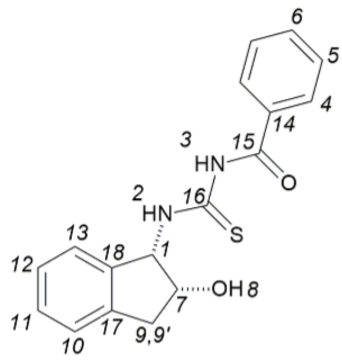

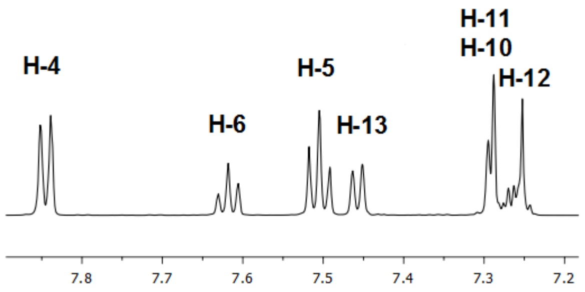

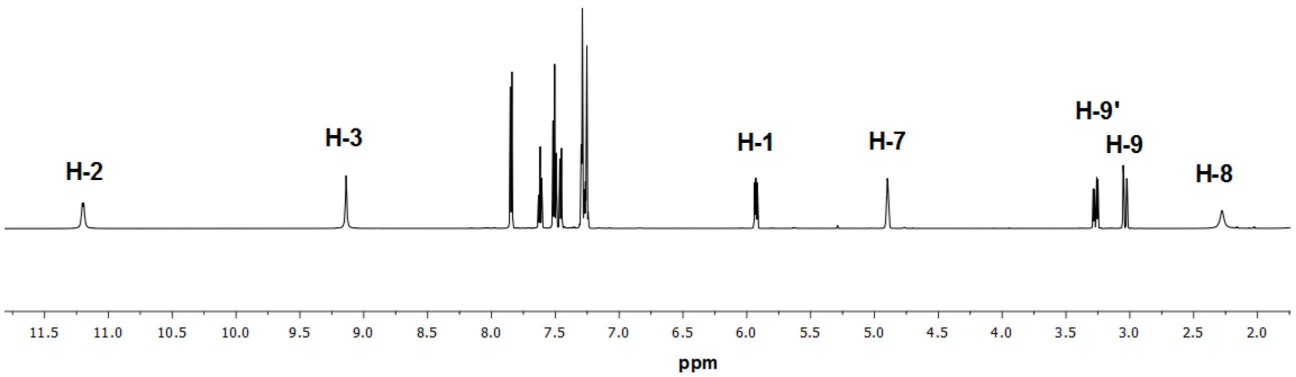

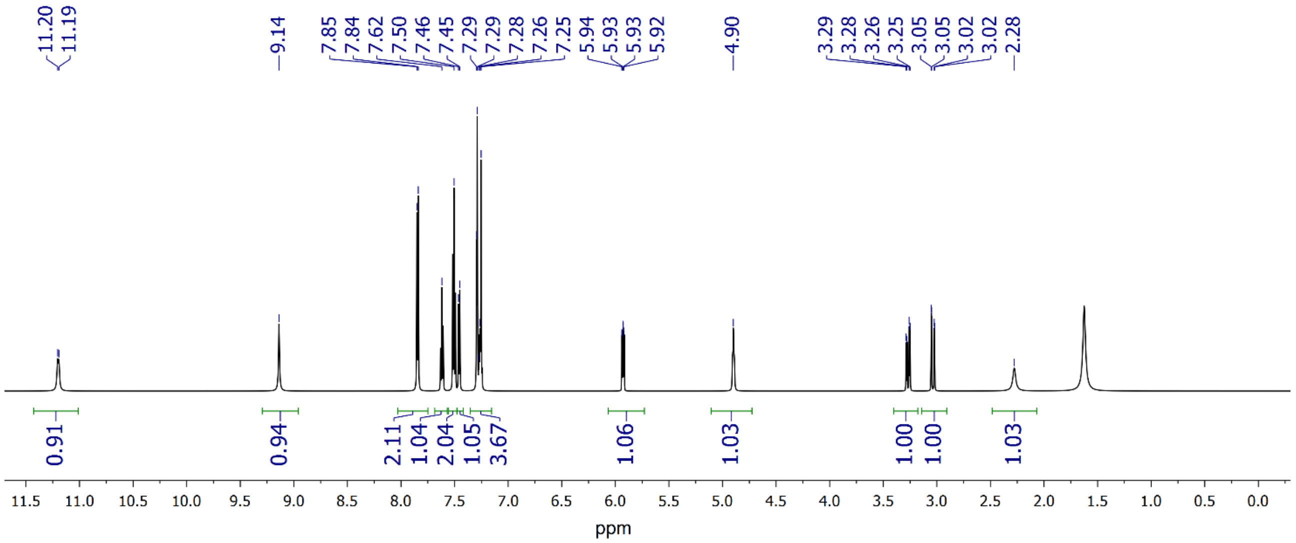

Figure S13. ^{1}H NMR (600 MHz, $CDCl_3$, 25 °C) spectrum of **2-TU**.

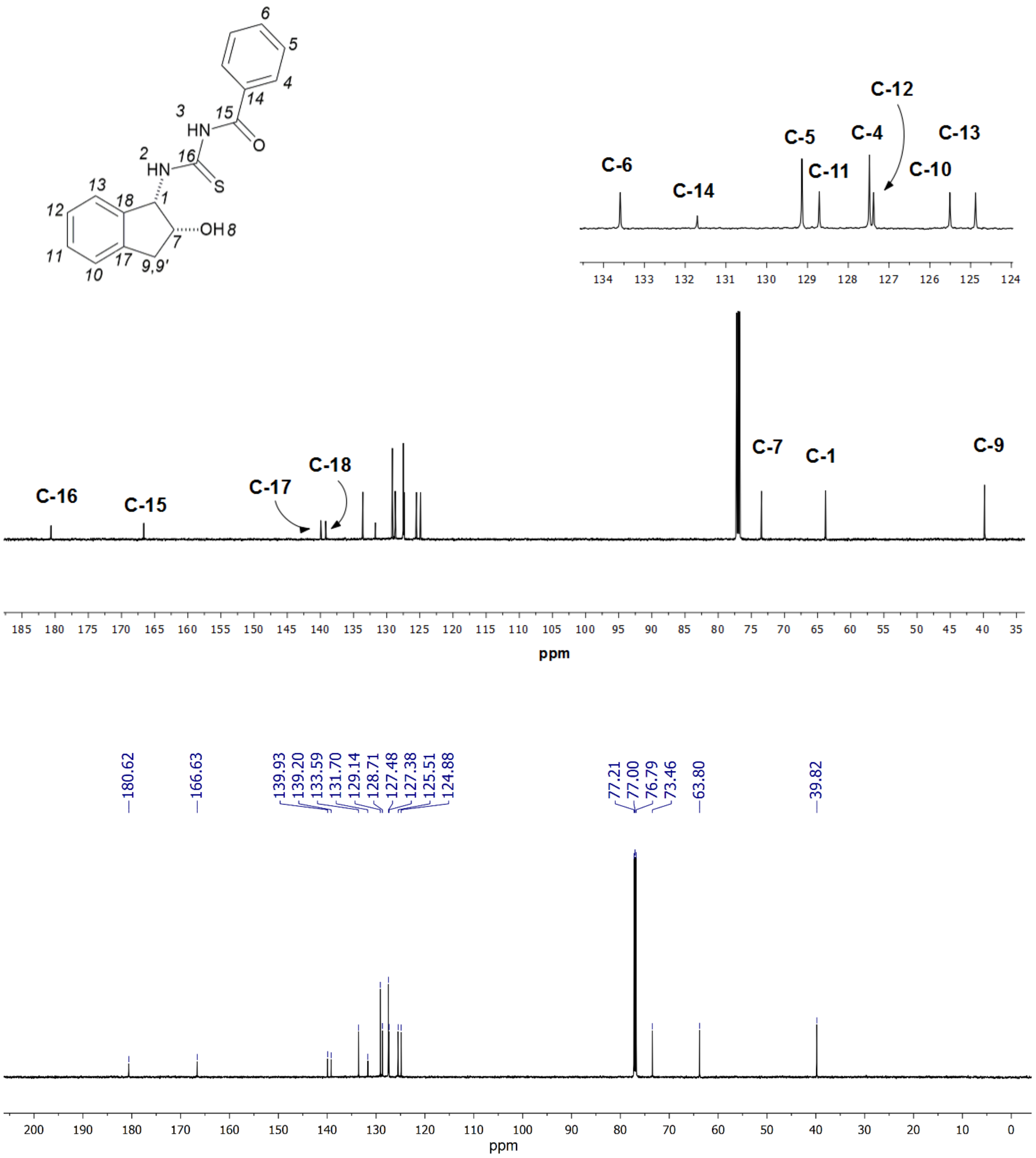

Figure S14. $^{13}C\{^{1}H\}$ NMR (150 MHz, $CDCl_3$, 25 °C) spectrum of **2-TU**.

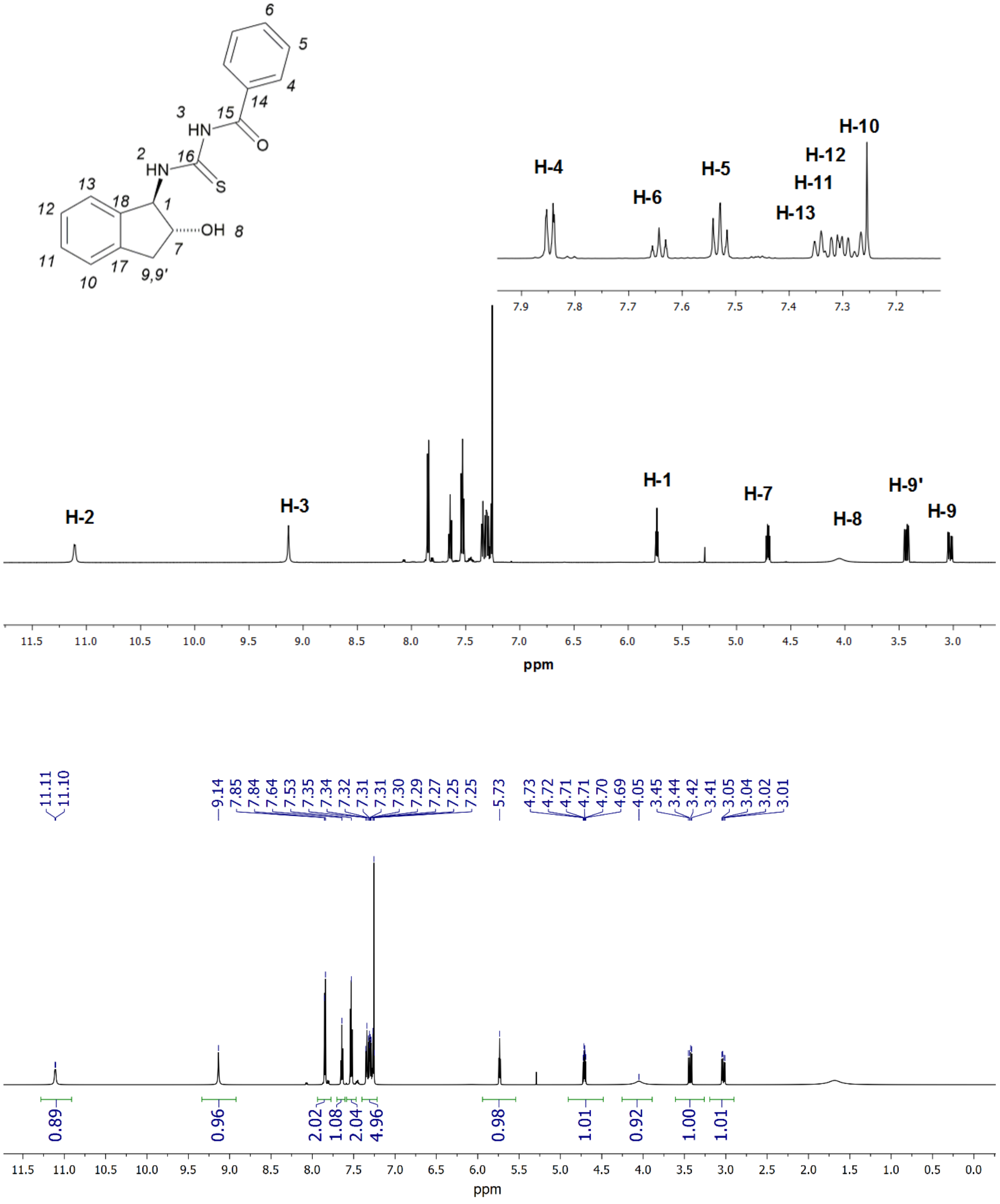

Figure S15. ^{1}H NMR (600 MHz, $CDCl_3$, 25 °C) spectrum of **3-TU**.

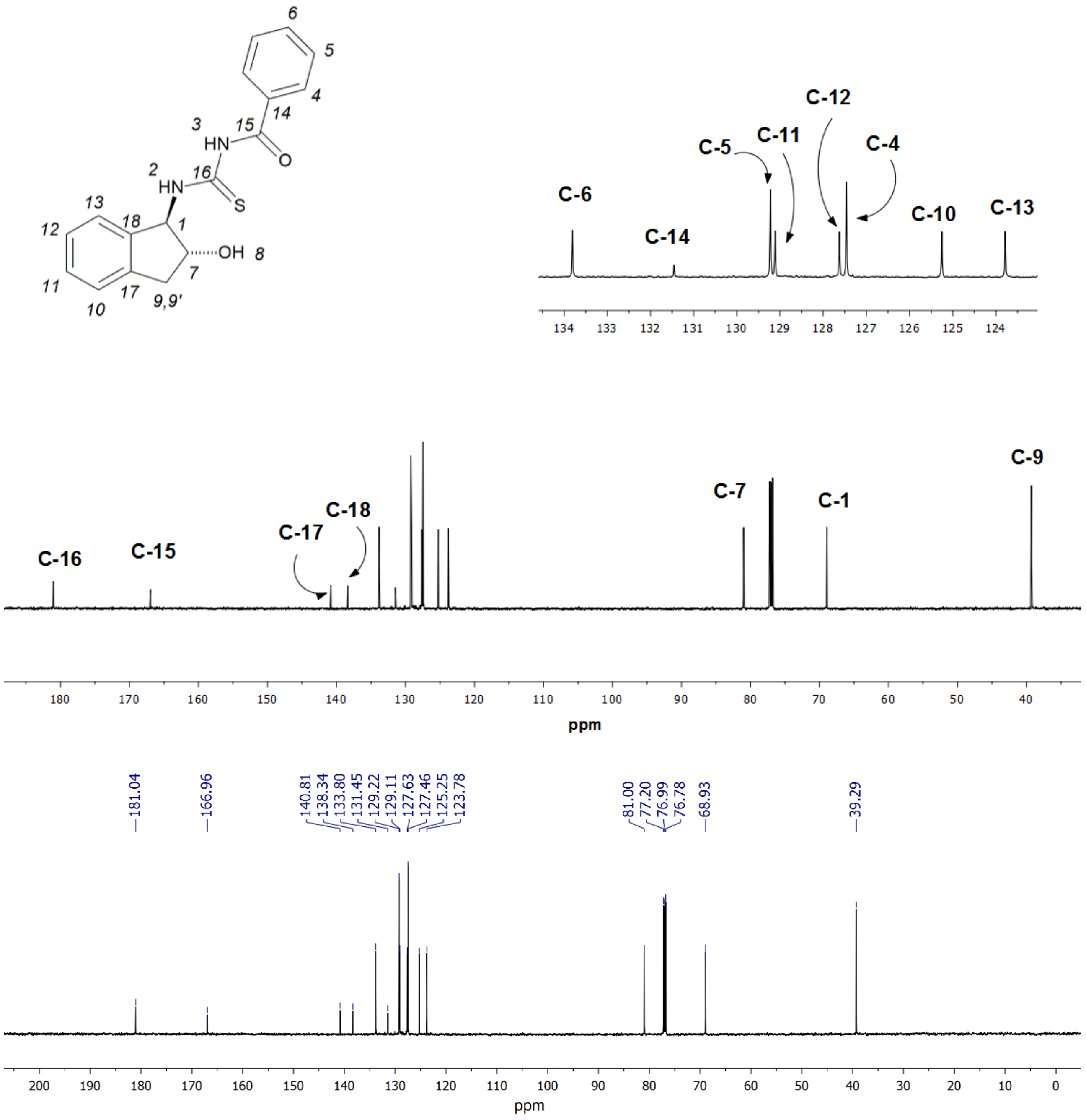

Figure S16. $^{13}C\{^{1}H\}$ NMR (150 MHz, $CDCl_3$, 25 °C) spectrum of **3-TU**.

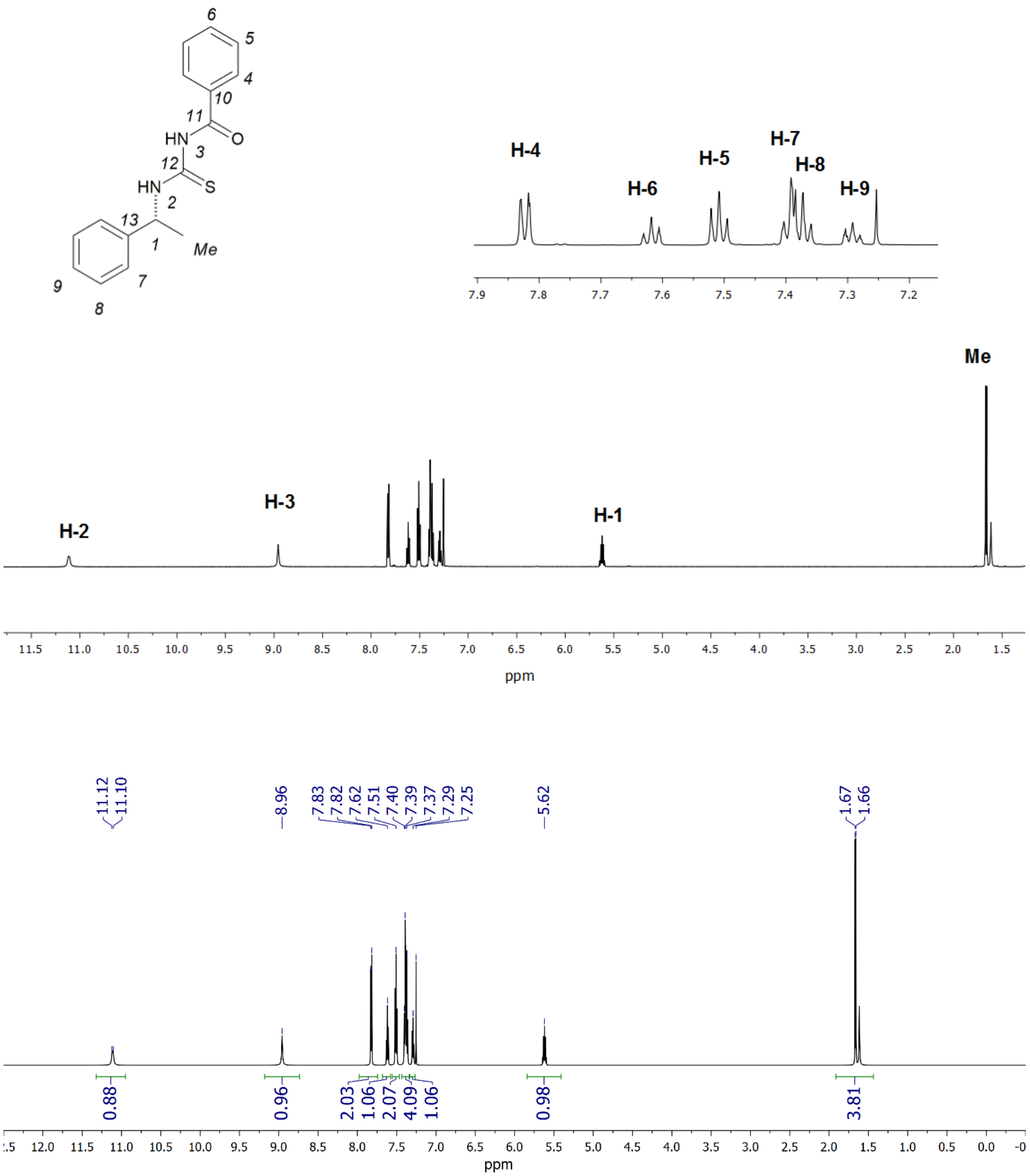

Figure S17. ^{1}H NMR (600 MHz, $CDCl_3$, 25 °C) spectrum of **4-TU**.

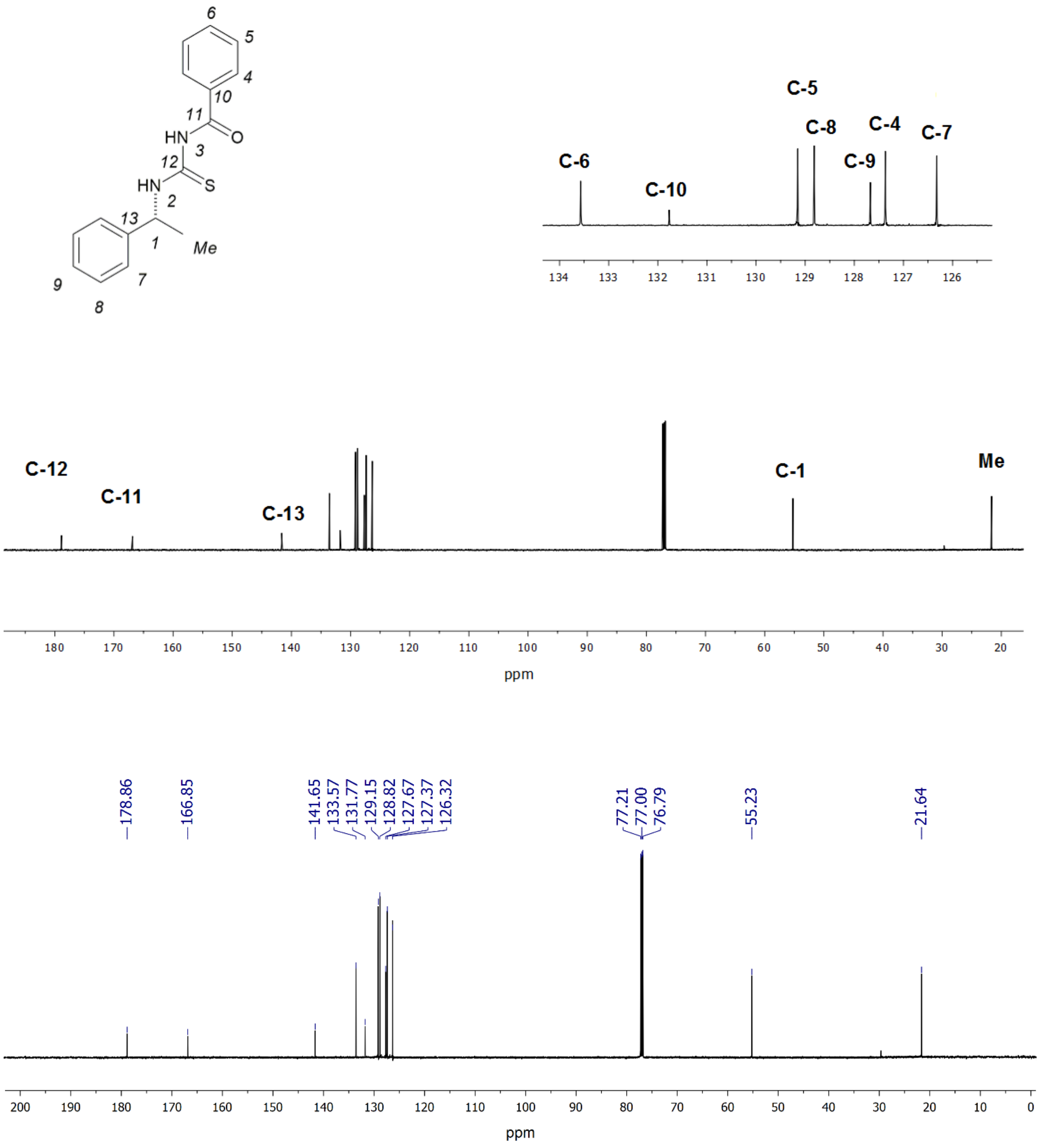

Figure S18. $^{13}C\{^{1}H\}$ NMR (150 MHz, $CDCl_3$, 25 °C) spectrum of **4-TU**.

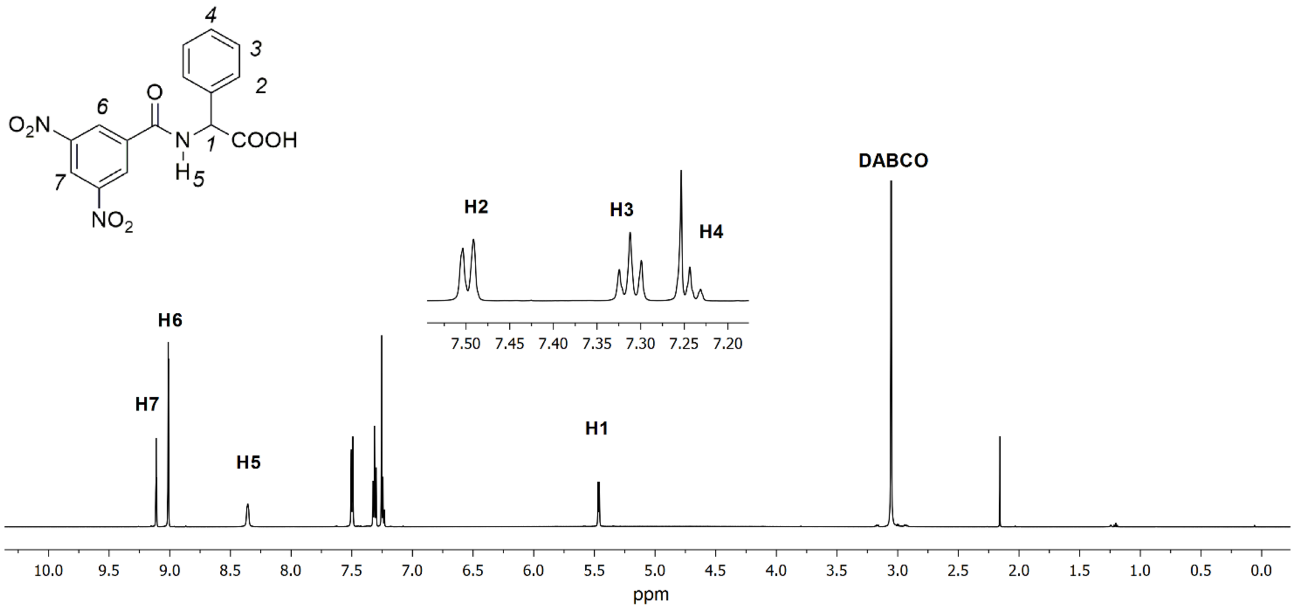

Figure S19. ^{1}H NMR (600 MHz, $CDCl_3$, 25 °C) spectrum of **5**.

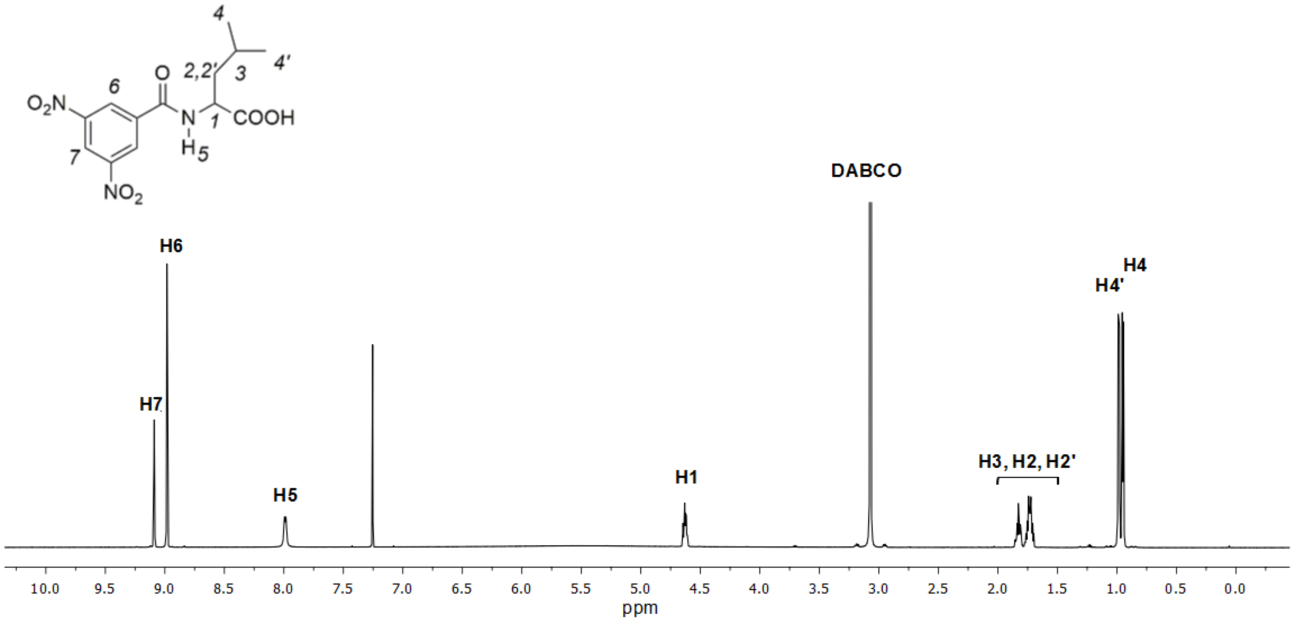

Figure S20. ^{1}H NMR (600 MHz, $CDCl_3$, 25 °C) spectrum of **6**.

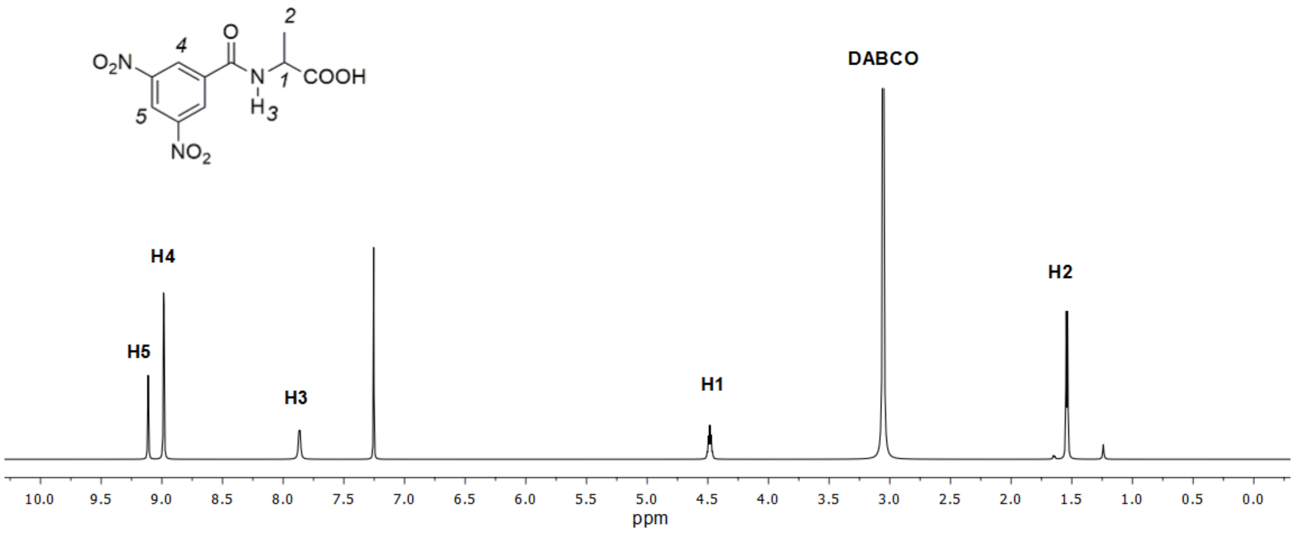

Figure S21. ^{1}H NMR (600 MHz, $CDCl_3$, 25 °C) spectrum of **7**.

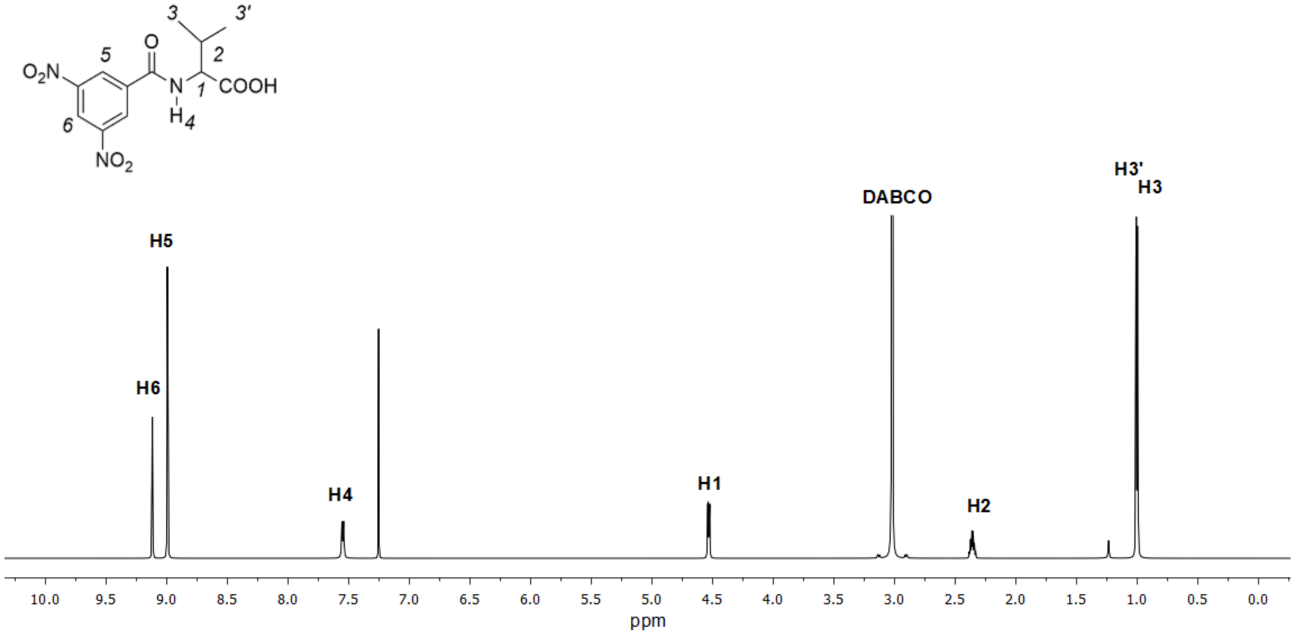

Figure S22. ^{1}H NMR (600 MHz, $CDCl_3$, 25 °C) spectrum of **8**.

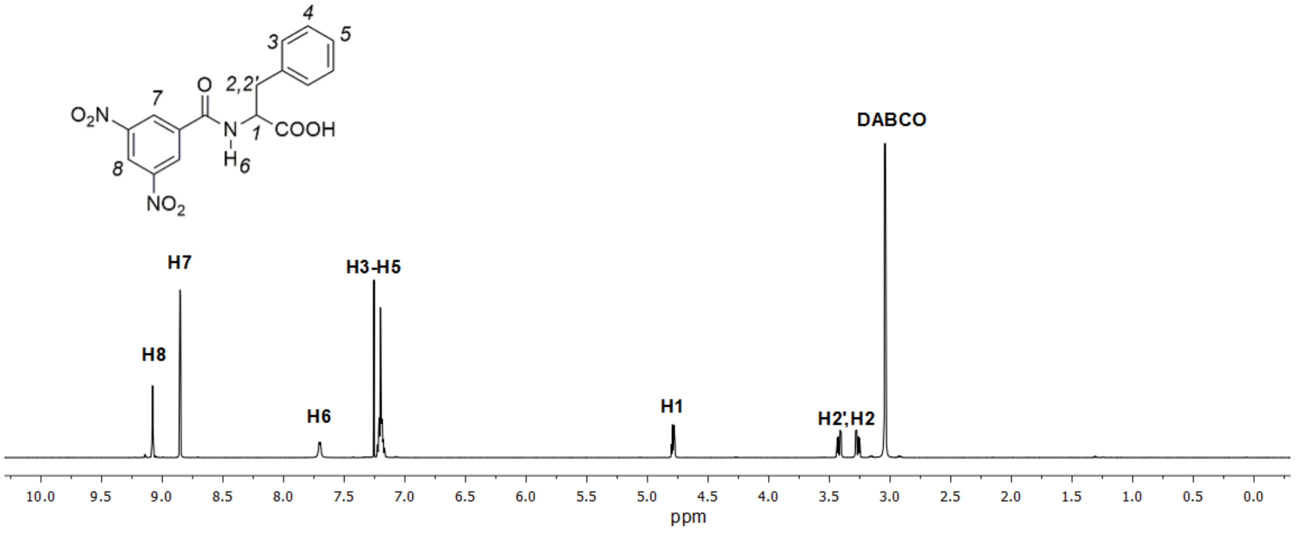

Figure S23. ^{1}H NMR (600 MHz, $CDCl_3$, 25 °C) spectrum of **9**.

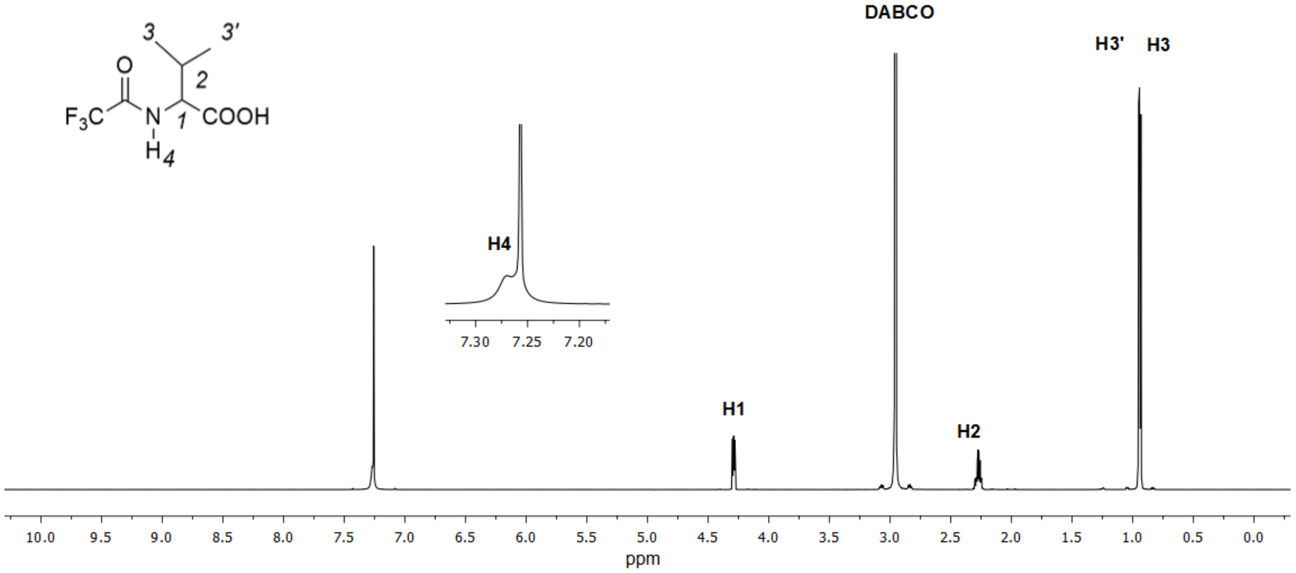

Figure S24. ^{1}H NMR (600 MHz, $CDCl_3$, 25 °C) spectrum of **10**.

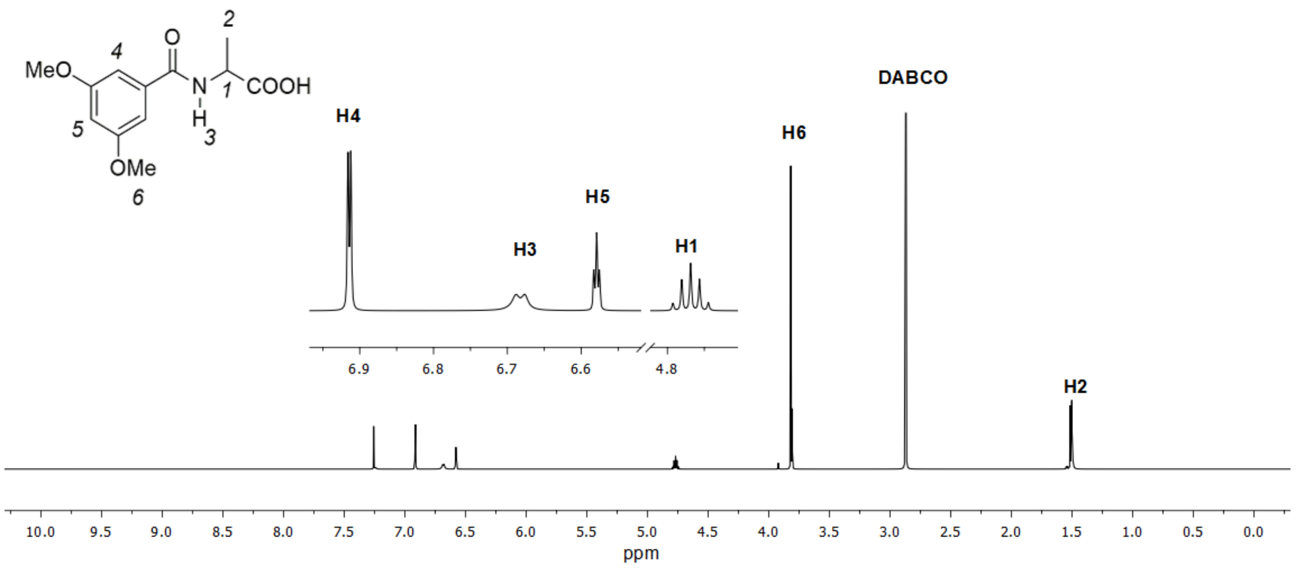

Figure S25. ^{1}H NMR (600 MHz, $CDCl_3$, 25 °C) spectrum of **11**.

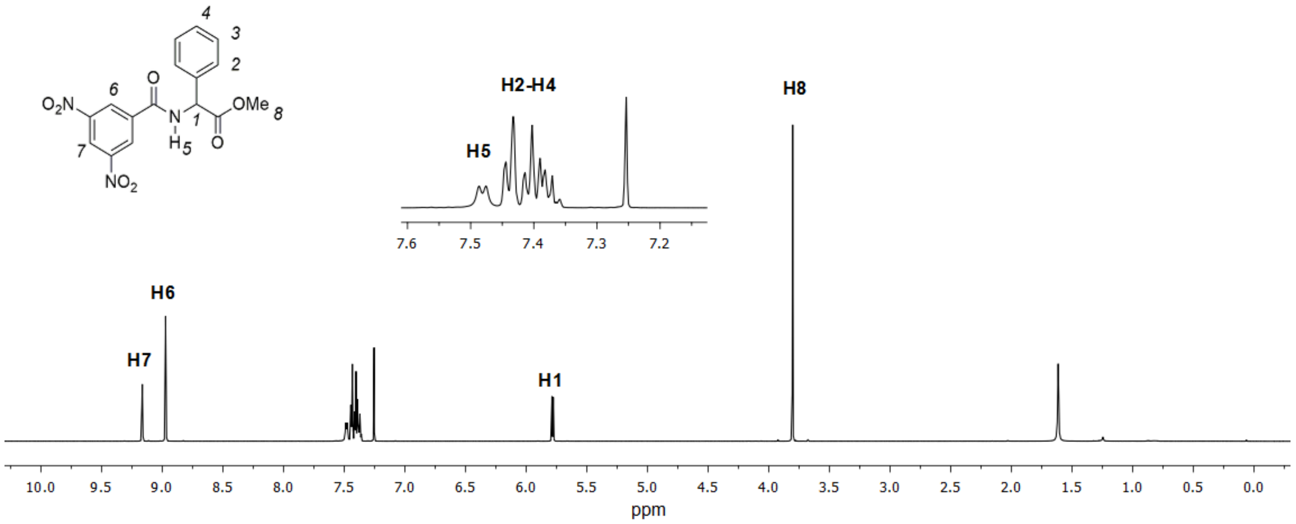

Figure S26. ^{1}H NMR (600 MHz, $CDCl_3$, 25 °C) spectrum of **12**.

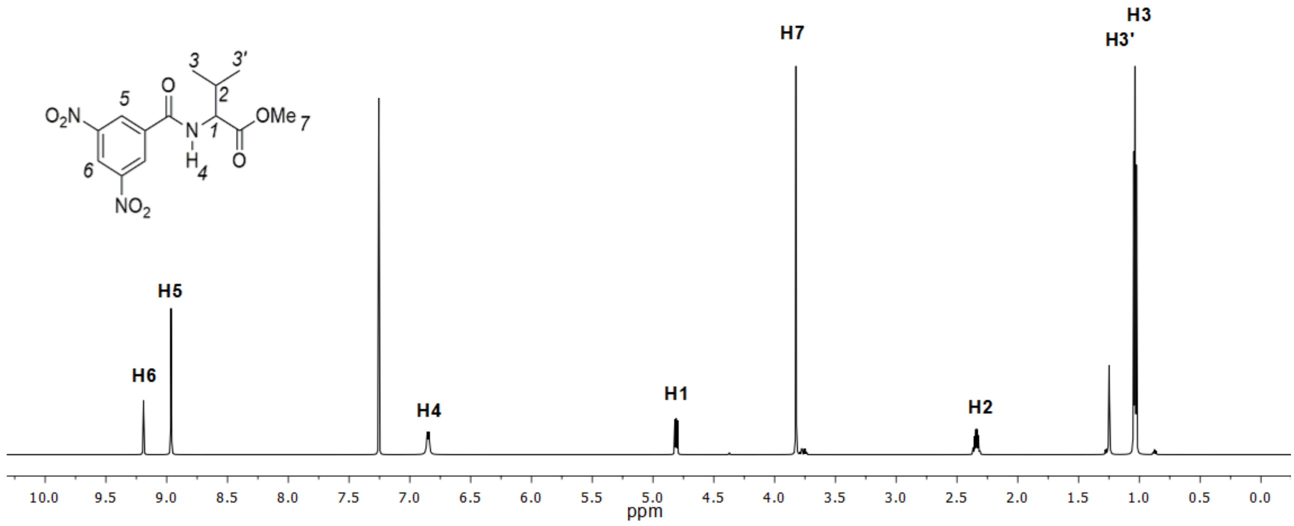

Figure S27. ^{1}H NMR (600 MHz, $CDCl_3$, 25 °C) spectrum of **13**.

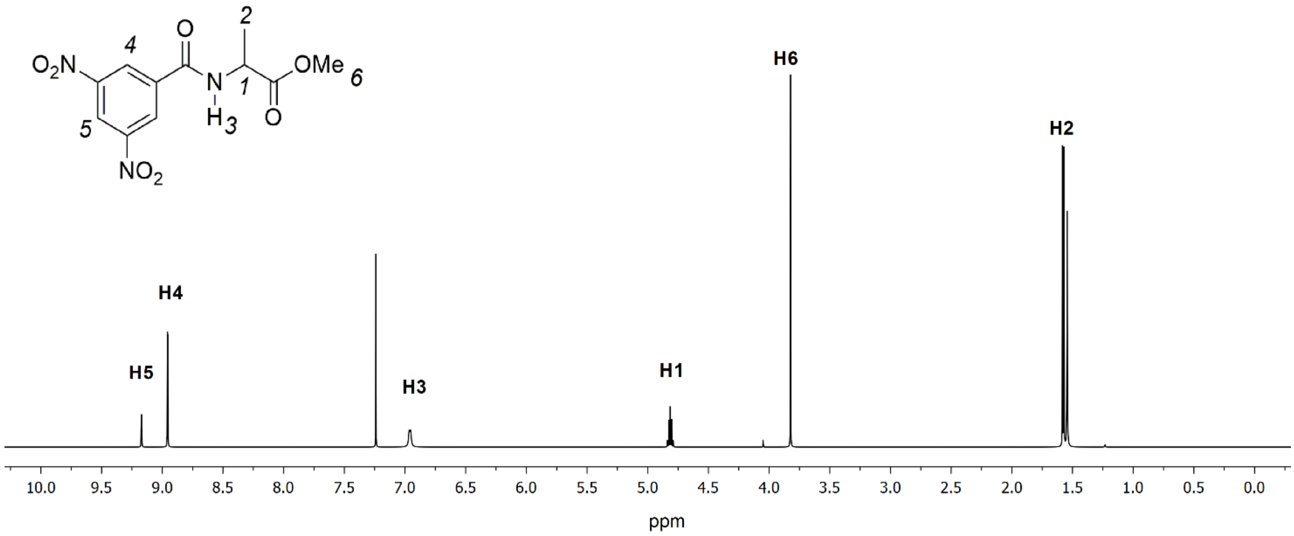

Figure S28. ^{1}H NMR (600 MHz, $CDCl_3$, 25 °C) spectrum of **14**.

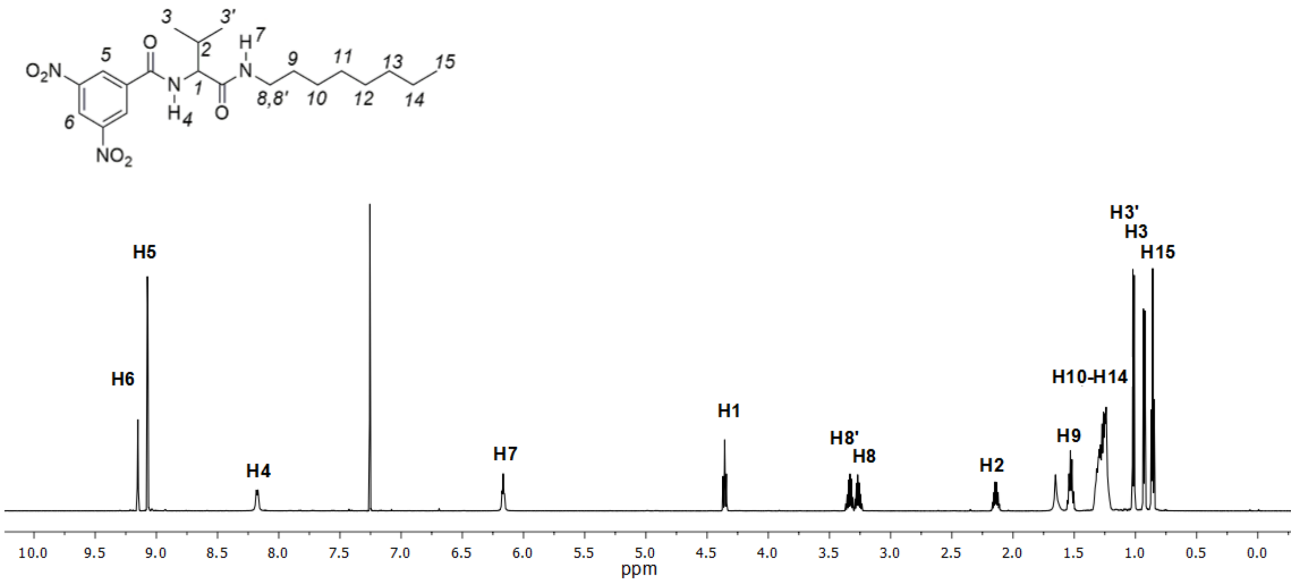

Figure S29. ^{1}H NMR (600 MHz, $CDCl_3$, 25 °C) spectrum of **15**.

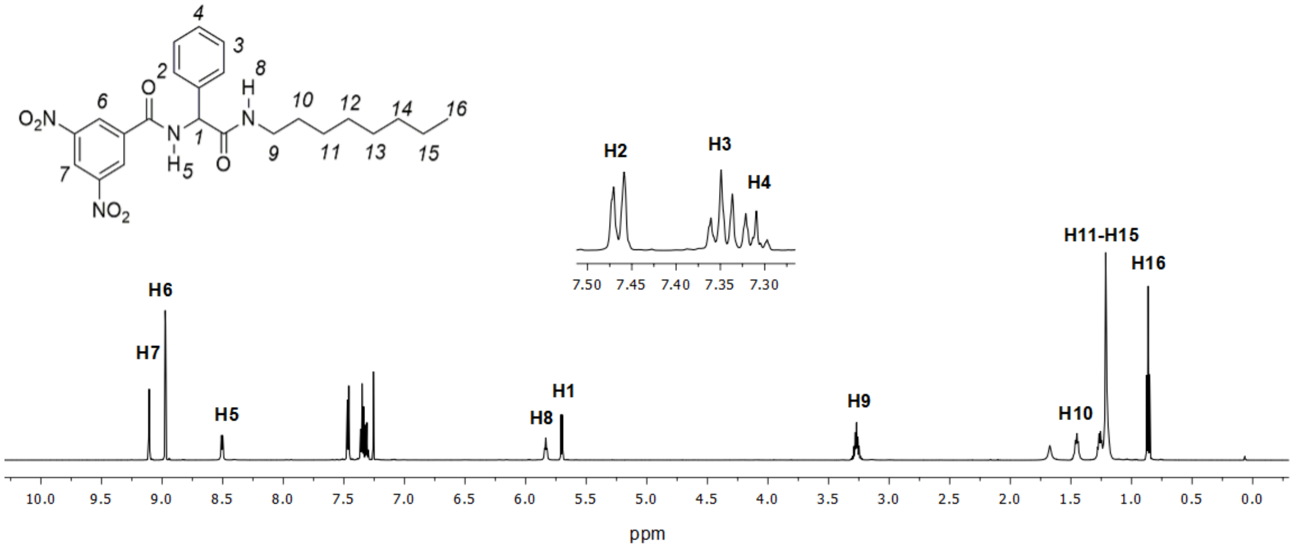

Figure S30. ^{1}H NMR (600 MHz, $CDCl_3$, 25 °C) spectrum of **16**.

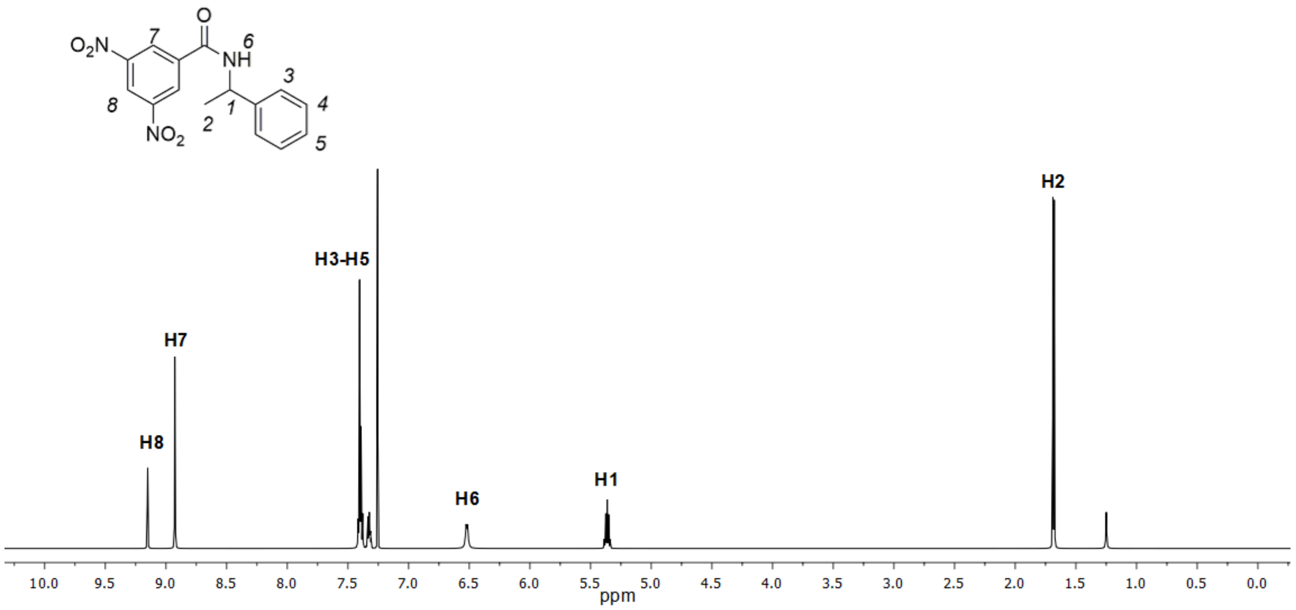

Figure S31. ^1H NMR (600 MHz, $CDCl_3$, 25 °C) spectrum of **17**.
